# A Novel Soluble Immune-Type Receptor (SITR) in Teleost Fish: Carp SITR Is Involved in the Nitric Oxide-Mediated Response to a Protozoan Parasite

**DOI:** 10.1371/journal.pone.0015986

**Published:** 2011-01-31

**Authors:** Carla M. S. Ribeiro, Steve Bird, Geert Raes, Gholamreza H. Ghassabeh, Virgil E. J. C. Schijns, Maria J. S. L. Pontes, Huub F. J. Savelkoul, Geert F. Wiegertjes

**Affiliations:** 1 Cell Biology and Immunology Group, Department of Animal Sciences, Wageningen University, Wageningen, The Netherlands; 2 Scottish Fish Immunology Research Centre, School of Biological Sciences, University of Aberdeen, Aberdeen, United Kingdom; 3 Laboratory of Cellular and Molecular Immunology, Vrije Universiteit Brussel, Brussels, Belgium; 4 Department of Molecular and Cellular Interactions, Vrije Universiteit Brussel, Brussels, Belgium; Charité-University Medicine Berlin, Germany

## Abstract

**Background:**

The innate immune system relies upon a wide range of germ-line encoded receptors including a large number of immunoglobulin superfamily (IgSF) receptors. Different Ig-like immune receptor families have been reported in mammals, birds, amphibians and fish. Most innate immune receptors of the IgSF are type I transmembrane proteins containing one or more extracellular Ig-like domains and their regulation of effector functions is mediated intracellularly by distinct stimulatory or inhibitory pathways.

**Methodology/Principal Findings:**

Carp SITR was found in a substracted cDNA repertoire from carp macrophages, enriched for genes up-regulated in response to the protozoan parasite *Trypanoplasma borreli*. Carp SITR is a type I protein with two extracellular Ig domains in a unique organisation of a N-proximal V/C2 (or I-) type and a C-proximal V-type Ig domain, devoid of a transmembrane domain or any intracytoplasmic signalling motif. The carp SITR C-proximal V-type Ig domain, in particular, has a close sequence similarity and conserved structural characteristics to the mammalian CD300 molecules. By generating an anti-SITR antibody we could show that SITR protein expression was restricted to cells of the myeloid lineage. Carp SITR is abundantly expressed in macrophages and is secreted upon *in vitro* stimulation with the protozoan parasite *T. borreli*. Secretion of SITR protein during *in vivo T. borreli* infection suggests a role for this IgSF receptor in the host response to this protozoan parasite. Overexpression of carp SITR in mouse macrophages and knock-down of SITR protein expression in carp macrophages, using morpholino antisense technology, provided evidence for the involvement of carp SITR in the parasite-induced NO production.

**Conclusion/Significance:**

We report the structural and functional characterization of a novel soluble immune-type receptor (SITR) in a teleost fish and propose a role for carp SITR in the NO-mediated response to a protozoan parasite.

## Introduction

The innate immune system is an ancient form of host defense that relies upon a wide range of non-rearranging, germ-line encoded receptors including a large number of immunoglobulin superfamily (IgSF) receptors [1], [2]. Members of the IgSF typically contain at least one Ig domain of about 100 amino acids built up by a sandwich of two β-sheets of antiparallel β-strands packed together and roughly forming a barrel-shaped structure. Ig domains are either of the variable (V) type, the constant (C)1 or C2 types or the intermediate (I) type differing by varying numbers of β-strands in each of the β-sheets that form the sandwich [3], [4]. The number and organization of the Ig domains in surface bound proteins of the IgSF may vary, but usually the N-terminal Ig domain is of the V-type, whereas the remaining domain(s) are of the C1 or C2-type [5], [6].

Well-studied Ig-like immune receptors comprise the leukocyte receptor cluster (LRC) on human chromosome 19 [7]. LRC genes can be grouped into different multigene families, which induce leukocyte-Ig-like receptors (LILRs), Ig-like transcripts (ILTs) [8], killer inhibitory receptors (KIRs) [9], platelet collagen receptor glycoprotein VI (GPVI) [10], receptor for IgAFc (FCAR) [11], natural cytotoxicity receptor (NCR) NKp46 [12] and leukocyte-associated inhibitory receptors (LAIRs) [13]. In addition to the LRC, two other small clusters have been identified in the human genome on chromosome 6 and 17. A cluster of single V-type Ig domain innate immune receptors on human chromosome 6 includes the natural cytoxicity receptor NKp44, triggering receptors expressed on myeloid cells (TREM) and TREM-like transcripts (TLT) [14], [15]. More distant relatives of TREM proteins are the CD300 family found on human chromosome 17, as well as the polymeric Ig receptor (pIgR). CD300 molecules are transmembrane glycoproteins with a single V-type Ig domain with a conserved YWCR amino acid motif and two (instead of one) disulfide bonds and an intracytoplasmic signaling motif [16], [17], [18]. Most innate immune receptors of the IgSF are type I transmembrane proteins containing one or more extracellular Ig-like domains, a transmembrane segment and a cytoplasmic region that may contain tyrosine residues [2]. Typically, their regulation of effector function is mediated intracellularly by distinct stimulatory or inhibitory pathways. Stimulatory receptors have a short cytoplasmic tail devoid of canonical signalling motifs but contain a positively charged amino acid residue within their transmembrane region that allows the receptor to associate with ITAM (immune receptor tyrosine-based activation motifs)-containing transmembrane adaptor proteins [19], [20]. Inhibitory receptors have long cytoplasmic tails with a variable number of ITIMs (immune receptor tyrosine-based inhibition motifs) [21], [22].

Soluble receptors can be generated by several mechanisms, which include proteolytic cleavage of receptor ectodomains, alternative splicing of mRNA transcripts or transcription of distinct genes that encode soluble receptors [23]. In mammals, LILRA3 and LAIR2 encoded within the LRC, are devoid of a transmembrane region and are secreted rather than embedded in the cell membrane [24], [25]. In addition, soluble forms of the TREM family members (TREM-1, TREM-2 and TLT-1) have been described [26], [27], [28]. Although their function is unknown, sTREM-1 and sTREM-2 are thought to negatively regulate TREM receptors signaling through neutralization of the respective ligands. Some reports suggest that soluble TREM are obtained by alternative splicing of mRNA transcripts whereas others report origination by proteolytic cleavage of the receptors' ectodomain [29].

Studies in non-mammalian vertebrates have reported the presence of Ig-like immune receptor families in birds, amphibians and fish [30]. Novel immune-type receptors (NITRs) are present in a large number of teleost fish species, are encoded by multigene families and share structural and signaling similarities with mammalian KIR receptors [31], [32]. Teleost novel immunoglobulin-like transcripts (NILTs) share structural similarities with mammalian TREM and NKp44 receptors [33], [34]. Modular domain immune-type receptors (MDIRs) from cartilaginous fish (clearnose skate) and zebrafish share structural similarities with mammalian CD300, TREM/TLT, FCAR and pIgR receptors [18], [35]. We report the structural and functional characterization of a Soluble Immune-Type Receptor (SITR) in a teleost fish. Carp SITR is a type I protein with two extracellular Ig domains in a rare organisation of a N-proximal V/C2 (or I-) type Ig domain and a C-proximal V-type, devoid of a transmembrane domain or any intracytoplasmic motif. The V-type Ig domain of SITR shows clear sequence homology to mammalian vertebrate CD300 molecules. Carp SITR is expressed abundantly in macrophages and can be secreted upon stimulation with the protozoan parasite *Trypanoplasma borreli*. Carp SITR promotes PKC-dependent NO production in the mouse macrophage RAW cell line and is involved in *T. borreli*-induced iNOS gene expression in carp macrophages.

## Results

### Cloning and sequence analysis of carp Soluble Immune-Type Receptor SITR

A substracted cDNA repertoire from common carp macrophages, enriched for genes up-regulated in response to the protozoan parasite *T. borreli*, was generated by SSH. Included in the repertoire (3/300 positive clones) was a partial consensus sequence for a novel immune-type receptor. Specific primers based on the initial sequence amplified a full-length cDNA sequence of 1114 bp with an open reading frame of 723 bp, encoding for a protein of 241 aa with a predicted molecular weight of 27.4 KDa.

**Figure 1:**
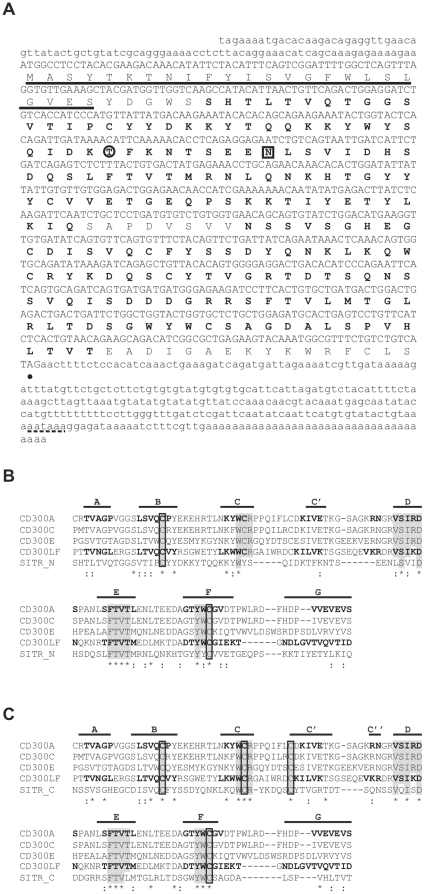
Carp Soluble Immune-Type Receptor (SITR) is a member of the Ig superfamily. A. Nucleotide sequence of common carp SITR with open reading frame (upper case) and untranslated 5′ and 3′ regions (lower case). The predicted amino acid sequence is shown below the nucleotide sequence. The predicted signal peptide is underlined and the two Ig-like domains are marked in bold. The potential *N*-glycosylation site is boxed and the potential PKC interaction site is circled. Dot indicates the stop codon. A consensus polyadenylation signal (AATAAA) in the 3′-UTR is dashed. B. Alignment of the putative carp SITR N-proximal Ig-like domain (SITR_N, residues 30–123) with V-type Ig domains from human CD300 molecules. C. Alignment of the putative carp SITR C-proximal Ig-like domain (SITR_C, residues 132–224) with V-type Ig domains from human CD300 molecules. Asterisks indicate identity and colons denote similarity. Dashes indicate the introduced gaps to maximize the alignment. Residues characteristic of the V-type CD300 Ig-like fold and conserved between carp SITR (GenBank Accession Number: HM370297, http://www.ncbi.nlm.nih.gov/genbank/) and human CD300A (GenBank acc no: NP_009192.2), CD300C (GenBank acc no: NP_006669.1), CD300E (GenBank acc no: NP_852114.1), CD300F (GenBank acc no: NP_620587.2) are grey shaded. Cysteines conserved between carp SITR and human CD300 molecules are boxed. Regions of β-strands, as defined by X-ray crystallography for CD300A (PDB acc no: 2Q87, http://www.rcsb.org/pdb/home/home.do) and CD300LF (PDB acc no: 2NMS) are marked in bold. The positions of the predicted β-strands for carp SITR are indicated above the sequence.

The novel cDNA has two predicted Ig-like domains and a 15 aa short proximal C-terminal region. Although the 15 aa proximal C-terminal region appears rich in positively charged amino acids (K232, K234, R236), the protein has no clear transmembrane region nor structural hallmarks suggestive of stimulatory or inhibitory (e.g.: ITAM, ITIM) signalling potential (Fig. 1A). The novel protein has a putative *N*-glycosylation site at position 73 and several Serine (13 sites), Threonine (4 sites) and Tyrosine (6 sites) phosphorylation sites as well as a protein kinase C (PKC) phosphorylation site at position T65 (Fig. 1A). Sequence analysis classifies it as a type I soluble protein with a putative hydrophobic 24-aa signal peptide expected to induce secretion. No vacuolar targeting signal was predicted. Thus, structural analysis of the novel protein sequence suggests SITR to be a secreted protein.

Amino acid sequence alignment of the two Ig-like domains (Fig. 1B–C) showed a high degree of similarity (BLAST *E* value≤10^−9^) of the C-proximal Ig-like domain (Fig. 1C, 93 aa) with Ig domains from mouse CMRF-35-like molecules (CLM) and human CD300 orthologues as well as with mammalian polymeric immunoglobulin receptor (pIgR) molecules. In contrast, the degree of similarity of the N-proximal Ig-like domain (Fig. 1B, 94 aa) with these mammalian Ig domains was low (BLAST *E* value≤10^−4^). Most CD300 Ig domains are characterized by the presence of two pairs of cysteine residues and typical aa motifs contained in strands forming the two β-sheets of the Ig domain. The novel protein has a C-proximal Ig-like domain with two pairs of cysteine residues and a conserved WCR motif in strand C and conserved FTV motif in strand E (Fig. 1C). In contrast, the N-proximal Ig-like domain has a single pair of cysteine residues only, with a conserved tryptophan (W) motif in strand C and a conserved FTVT motif in strand E (Fig. 1B). Prediction of the β-strands using Swiss Model and PSIPRED databases and sequence alignment with β-strand regions defined by X-ray crystallography of CD300A and CD300 LF molecules, defined a long spacing between the putative β-strands C and D, suggesting the presence of additional β-strands (e.g.: C′ and C″). The prediction servers confirmed the presence of a C′ strand for both Ig-like domains and possibly a C″ strand for the C-proximal Ig-like domain. Thus, we predict the N-proximal Ig-like domain to be of the V/C2- (or I-) type and predict the C-proximal Ig-like domain to be of the V-type.

In conclusion, sequence analysis suggests that the novel cDNA is a new member of the Ig-SF with a V/C2 type N-proximal Ig domain and a V-type C-proximal Ig domain. The V-type Ig domain displays homology with mammalian CD300 molecules and with modular domain immune type receptors (MDIRs) of the cartilaginous skate and zebrafish which are multigenic families of activating/inhibitory receptors. We conclude that we have identified, in common carp, a novel soluble immune-type receptor without typical activating/inhibiting characteristics named Soluble Immune-Type Receptor SITR (GenBank Accession Number: HM370297, http://www.ncbi.nlm.nih.gov/genbank/).

### SITRs belong to a multigene family of soluble receptors

**Figure 2:**
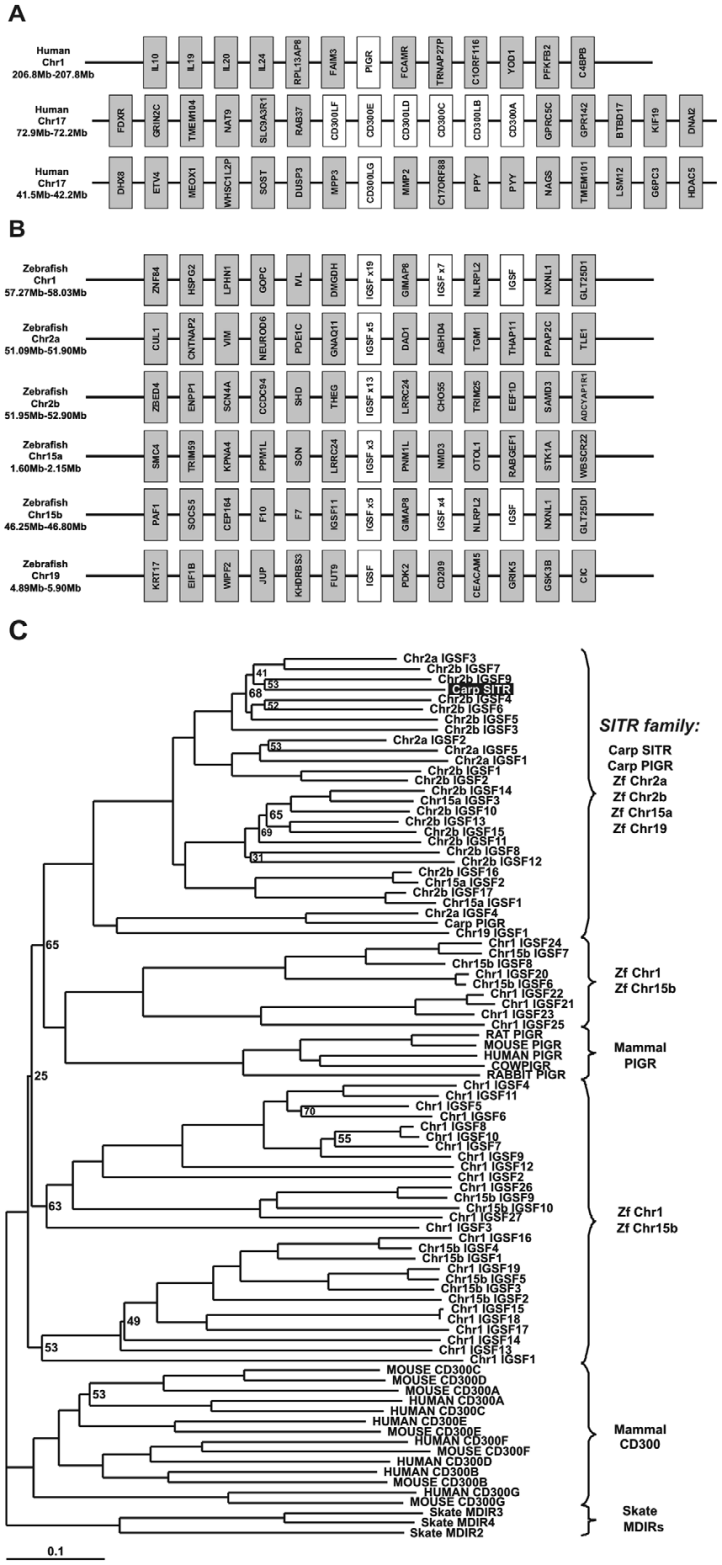
Diversity of carp SITR-related molecules encoded in the zebrafish genome. A. Schematic organization of human CD300 and PIGR loci on chromosomes (Chr) 17 and chromosome 1. B. Schematic organization of zebrafish SITR loci identified on chromosome 1, chromosome 2 (sites 2a and 2b), chromosome 15 (sites 15a and 15b) and chromosome 19. C. Unrooted phylogenetic tree showing the relationship between the carp SITR amino acid sequences for the full-length molecule with other known vertebrate Ig-like receptor sequences. This tree was constructed by the ‘neighbour-joining’ method using the clustal X and treeview packages, and was bootstrapped 10,000 times. All bootstrap values less than 75% are shown. The GenBank accession numbers (http://www.ncbi.nlm.nih.gov/genbank/) of the human CD300 amino acid sequences are: CD300A, NP_009192.2; ; CD300B, NP_777552.2 ; CD300C, NP_006669.1 ; CD300D, NP_001108624.1; CD300E, NP_852114.1; CD300F, NP_620587.2 ; CD300G, NP_660316.1. The GenBank accession numbers (http://www.ncbi.nlm.nih.gov/genbank/) of the mouse CD300 amino acid sequences are: CD300A, CAM18755.1; CD300B, NP_954691.2 ; CD300C, NP_954695.1; CD300D, NP_663412.1 ; CD300E, NP_742047.1; CD300F, NP_663609.2; CD300G, NP_082263.2. The GenBank accession numbers (http://www.ncbi.nlm.nih.gov/genbank/) of the skate sequences are: MDIR2, ABC86796.1; MDIR3, ABC86797.1; MDIR4, ABC86799.1. The GenBank accession numbers (http://www.ncbi.nlm.nih.gov/genbank/) of the mammalian PIGR are: RAT, NP_036855.1; MOUSE, NP_035212.2; HUMAN, NP_002635.2; COW, NP_776568.1; RABBIT, NP_001164516.1. The Genbank accession numbers (http://www.ncbi.nlm.nih.gov/genbank/) of the carp sequences are: PIGR, ADB97624.1; SITR, HM370297.

To investigate if the novel SITR gene could be part of a multigenic family we used the common carp SITR cDNA sequence to search for SITR orthologues in the genome of zebrafish, a close relative of common carp [36]. This search identified 6 sites coding for SITR-related molecules (named IGSF in Fig. 2B) in the zebrafish genome, of which several with multiple genes. We identified one site on zebrafish chromosome 1, two sites on chromosome 2 (2a, 2b), two sites on chromosome 15 (15a, 15b) and one site on chromosome 19. We could identify no synteny between mammalian CD300 or pIgR sites and SITR-related sites in zebrafish (Fig. 2 A–B).

Carp SITR and the SITR-related zebrafish sequences found on chromosome sites 2a, 2b, 15a and 19 form a cluster of soluble receptors only, without evidence for transmembrane domains or evidence for activating (ITAM) or inhibiting (ITIM) motifs (Fig. 2C). For this reason, we refer to this cluster as SITR-family. Additional SITR-related zebrafish sequences found on other chromosomal sites 1 and 15b do show evidence of transmembrane domains and form two multigene families distinct from the SITR-family. Taken together these results suggest carp SITR to be an orthologue of a multigenic family of SITRs in zebrafish.

### Carp SITR displays a high basal expression

**Figure 3:**
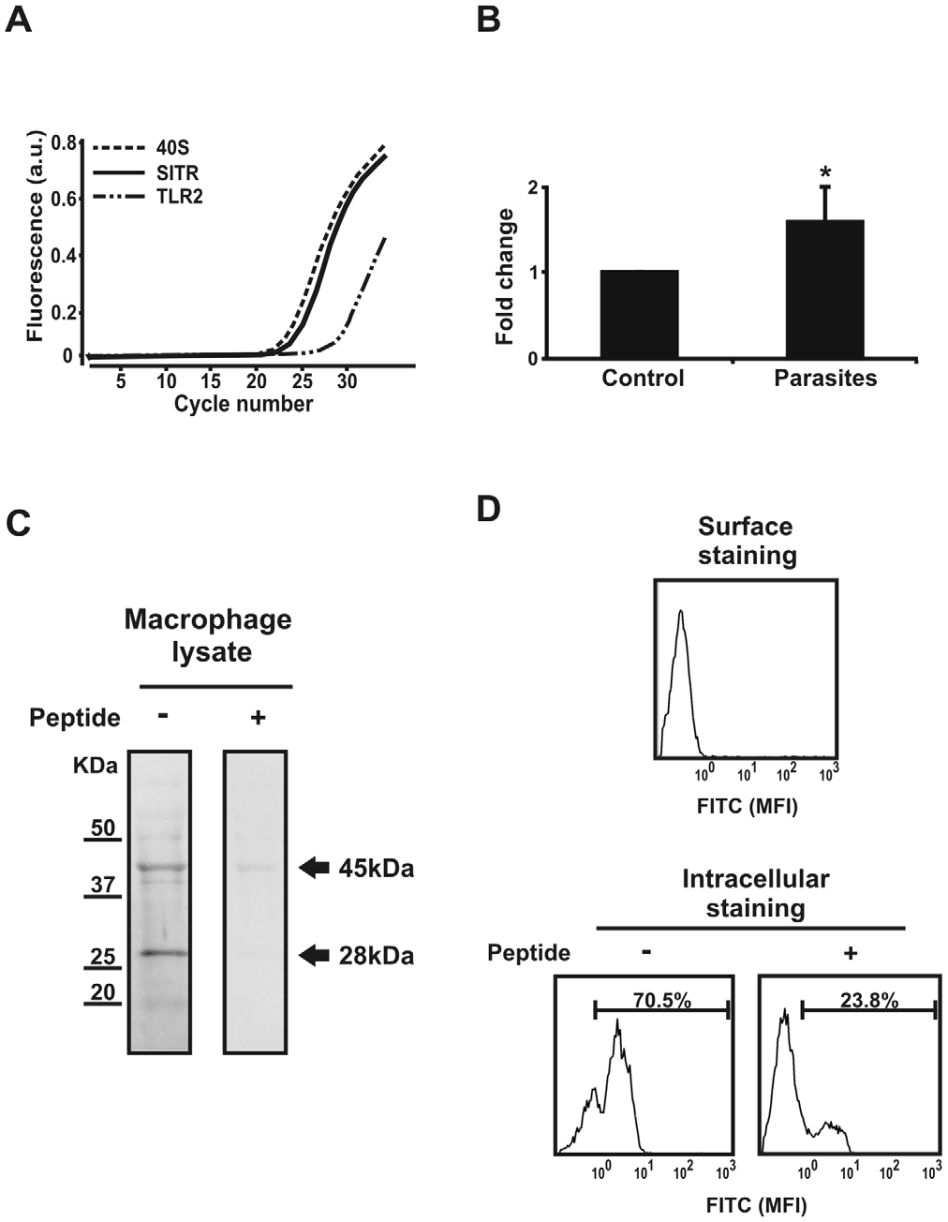
SITR gene and protein expression in carp macrophages. A. Real-time qPCR cycle profile for SITR in naïve carp macrophages in comparison with the house keeping gene 40S ribosomal protein S11 and Toll-Like Receptor (TLR)2 as reference genes. B. SITR gene expression in carp macrophages after stimulation for 6 h with live *T. borreli* protozoan parasites (0.5×10^6^ per well). mRNA levels of SITR relative to the house keeping gene 40S ribosomal protein S11 are expressed as fold change relative to unstimulated cells (control). Bars show averages ± SD of *n* = 4 fish. Symbol (*) shows a significant (*P*≤0.05) difference compared with unstimulated cells. C. Western blot analysis of macrophage lysates using as primary antibody the anti-SITR antibody or the anti-SITR antibody pre-incubated with the immunizing peptide (20µg/ml). D. Surface and intracellular SITR protein staining detected by flow cytometry using anti-SITR antibody or anti-SITR antibody pre-incubated with the immunizing peptide (20µg/ml).

The SITR cDNA was initially identified in an SSH repertoire from carp macrophages stimulated with the protozoan parasite *T. borreli*. Studies of basal SITR gene expression in carp macrophages revealed a highly abundant expression in naïve macrophages similar to the level of the house keeping 40S ribosomal S11 protein gene (Fig. 3A). Gene expression in carp macrophages could be 2-fold upregulated, approximately, by the protozoan parasite *T. borreli* (Fig. 3B).

To study protein expression, we affinity-purified an antibody raised in rabbit against a peptide in the C-proximal Ig-domain of SITR (YTVGRTDTSQNSSVQIS). Two discrete bands could be shown in western blot, of which one with predicted 28 KDa MW (Fig. 3C). Pre-incubation of the anti-SITR antibody with the immunizing peptide resulted in a partial disappearance of the 45KDa but complete disappearance of the band with predicted molecular weight of 28 KDa of SITR, confirming specificity of the antibody.

Sequence analysis showed that, although the 15 aa proximal C-terminal region of SITR appears rich in positively charged amino acids, the protein has no clear transmembrane region and has a secreted function. To study the sublocalization of the protein in carp macrophages, we performed both surface and intracellular staining using the anti-SITR antibody. Lack of surface staining (Fig. 3D) supports the sequence analysis that carp SITR has no transmembrane region nor plasma membrane anchoring by, for example, GPI modifications. Clear intracellular staining (Fig. 3D) of 50–70% of cells indicated that the majority of naïve carp macrophages express the SITR protein. As a negative control anti-SITR antibody was pre-incubated with the immunizing peptide, which reduced intracellular staining to 20% only. Our results indicate that carp SITR is a soluble immunoglobulin-type receptor abundantly expressed intracellularly in naïve carp leukocytes.

### Carp SITR is expressed mainly in myeloid cells

**Figure 4:**
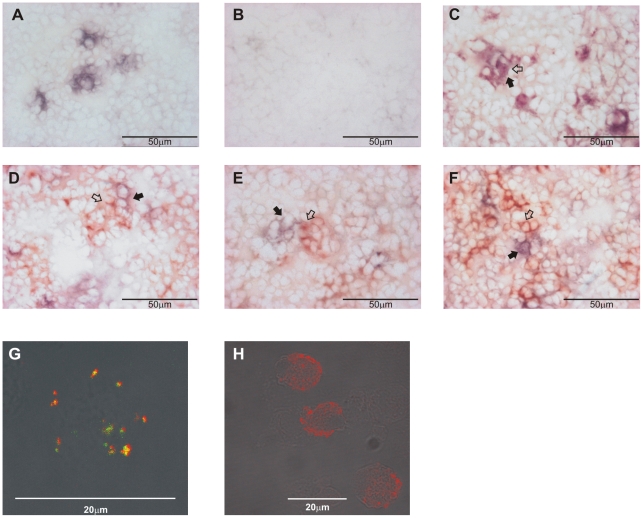
SITR protein is mainly expressed in myeloid cells. A. Anti-SITR immunoreactivity (blue) in spleen of naïve carp. B. Anti-SITR immunoreactivity (blue) after pre-incubation of the anti-SITR antibody with the immunizing peptide (20µg/ml) in spleen of naïve carp. C. Double-staining for monocytes/macrophages (WCL-15; red) and SITR (blue) in spleen of naïve carp. D. Double-staining for neutrophilic granulocytes (TCL-BE8; red) and SITR (blue) in spleen of naïve carp. E. Double-staining for B cells (WCI-12; red) and SITR (blue) in spleen of naïve carp. F. Double-staining for thrombocytes (WCL-6; red) and SITR (blue) in spleen of naïve carp. G. Double-staining for monocytes/macrophages (WCL-15; green) and SITR (red) in macrophage-enriched cell fractions from head-kidney of naïve carp. H. Staining for SITR (red) in macrophage-enriched cell fractions from head-kidney of naïve carp. Typical red-stained (WCL-15^+^, TCL-BE8^+^, WCI-12^+^ or WCL-6^+^) cells are indicated with open arrows and typical blue-stained (SITR^+^) cells with closed arrows. Note that in panel C, it is difficult to distinguish between red- and blue-stained cells. Co-localization of both signals results in the indicated dark purple-stained cells.

The SITR gene was detected in protozoan parasite-stimulated carp macrophages, whereas the presence of SITR protein in carp macrophages was confirmed by western blot and flow cytometry. To examine the putative presence of SITR protein in leukocyte cell types other than macrophages we performed double-staining using anti-SITR antibody (blue) in combination with monoclonal antibodies (red) recognizing (WCL-15+) monocytes/macrophages, (TCL-BE8+) neutrophilic granulocytes, (WCI-12+) B-cells or (WCL-6+) thrombocytes in spleen from naïve carp. Immunohistochemical analysis confirmed the abundant SITR protein expression particularly in splenic macrophages (WCL-15+ SITR+ double-positive cells display a dark-purple colour) but not in neutrophilic granulocytes, B cells and thrombocytes (Fig. 4A–F). To examine the putative presence of the SITR gene in leukocyte cell types other than macrophages we also performed real-time qPCR on cDNA from purified leukocyte cell populations. Gene expression analysis confirmed the abundant expression of SITR transcript in monocyte/macrophages, showed moderate SITR gene expression in neutrophilic granulocyte-enriched fractions and weak SITR gene expression in B cell-, T cell- and thrombocyte-enriched fractions (data not shown). This suggests that SITR is preferentially expressed in myeloid cell types. To verify the high SITR prevalence in macrophages, two further sources of carp macrophages were examined for SITR protein expression; macrophage-enriched MACS-sorted leukocytes from head kidney and head kidney-derived cultured macrophages. Head kidney is the hematopoietic organ equivalent to the mammalian bone marrow. WCL-15+ macrophages displayed SITR+ positivity (Fig. 4G–H). The SITR protein was never detected on the cell surface membrane but localized intracellularly within macrophages in vesicle-like structures.

### Carp SITR is secreted upon in vitro stimulation with protozoan parasites

**Figure 5:**
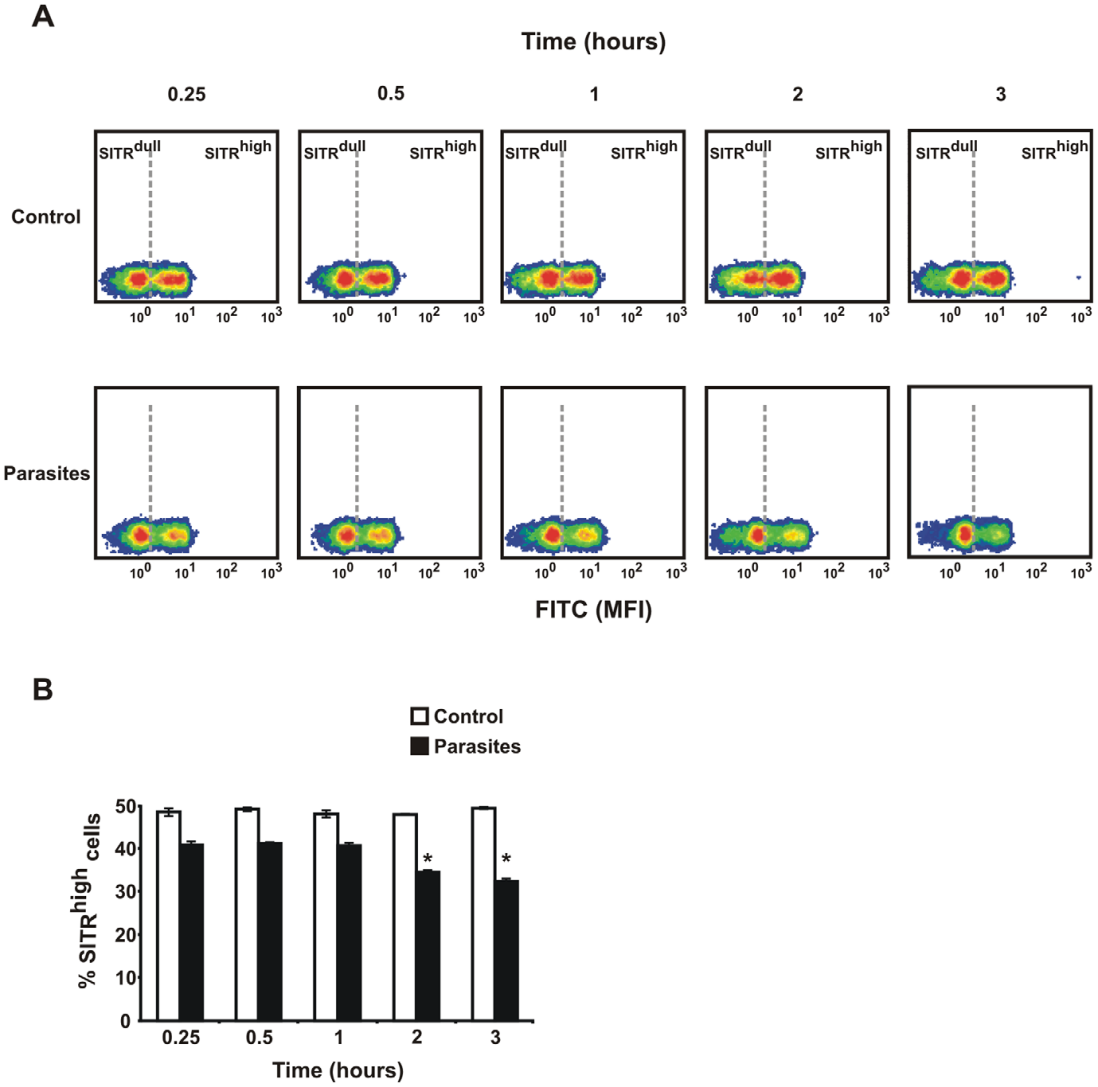
Effect of parasite stimulation on percentage of SITR-positive cells. A. Density plots of intracellular SITR protein expression analysed by flow cytometry using anti-SITR antibody. Macrophages were stimulated with live *T. borreli* parasites (0.5×10^6^ per well) for different time periods or left untreated as negative control. Two populations of cells could be defined on the basis of SITR protein expression: SITR^dull^ and SITR^high^ for the density plot representing negative control cells at 0.25 h. The separation (grey dashed line) between SITR^dull^ and SITR^high^ gates was defined based on each negative control (at 0.25, 0.5, 1, 2 and 3 hours) and set as the line separating the two populations. The same SITR^dull^ and SITR^high^ gate settings were used to analyse the parasite-stimulated samples at the respective time points. Density plots shown are representative of one out of three experiments. Mean fluorescent intensity (MFI) of FITC and PE are represented in X and Y axes, respectively. B. Percentage SITR^high^ cell populations (averages ± SD of *n* = 3 fish) after stimulation of macrophages with live *T. borreli* parasites for different time periods, or left untreated as negative control. Symbol (*) indicates a significant (*P*≤0.05) difference in parasite-stimulated cells compared with unstimulated cells at the same time point.

The SITR cDNA was initially identified to be differentially expressed in carp macrophages stimulated with *T. borreli*. To confirm SITR protein regulation by this protozoan parasite, macrophages were incubated with live *T. borreli* parasites. The presence of two distinct populations (SITR^dull^ and SITR^high^), corresponding to differences in SITR protein expression, was evident (Fig. 5A). Stimulation with live parasites did not have an effect on mean fluorescence intensity (MFI) but did result in a lower number of SITR^high^ cells after 15 min (0.25 hour; Fig. 5A). After 3 h stimulation with live parasites, the percentage of SITR^high^ macrophages reduced from 50% to 35%, approximately (Fig. 5B). Decrease of the percentage of SITR^high^ macrophages was evident after stimulation with the protozoan parasite *Trypanoplasma borreli* as well as after stimulation with a related parasite (*Trypanosoma carassii*), but not after stimulation with unrelated ligands such as peptidoglycan or lipopolysaccharide (data not shown). Our results therefore suggest a ligand-specific function for SITR in carp macrophages.

**Figure 6:**
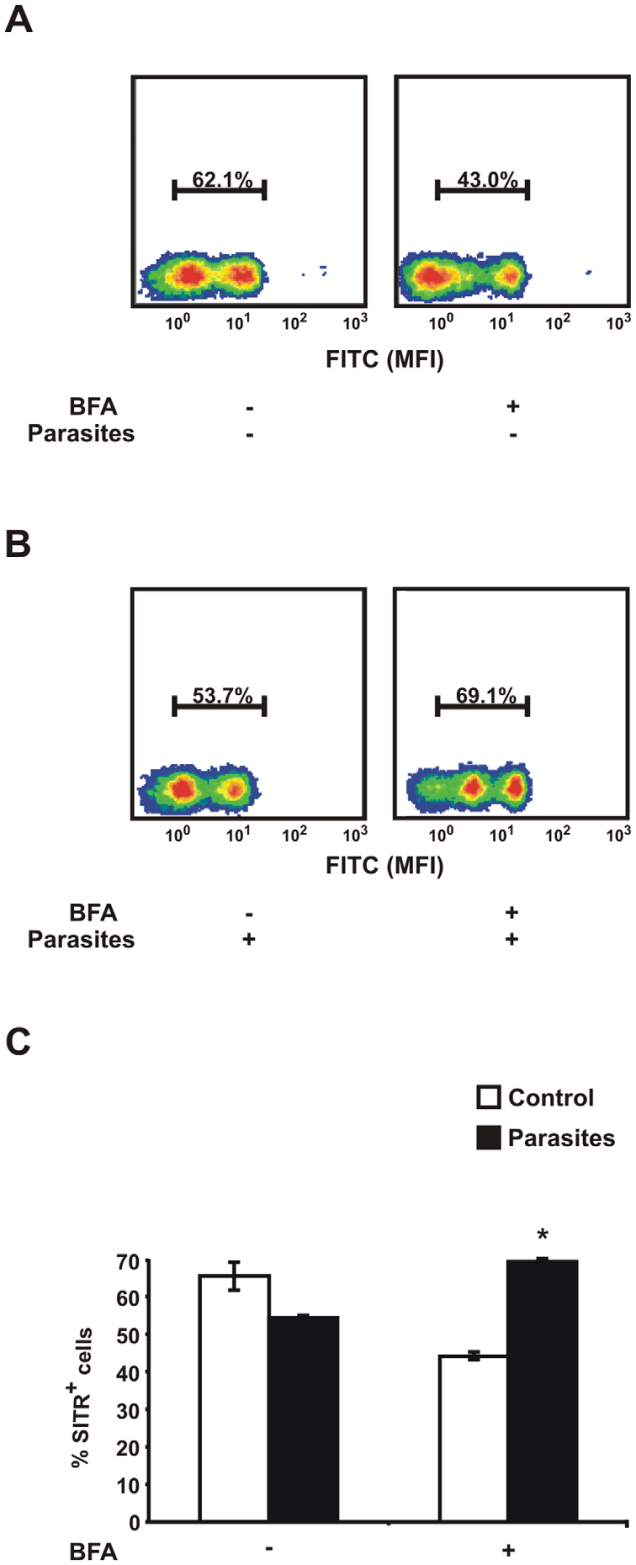
Intracellular SITR protein sorting. A. Carp macrophages were incubated for 16.5 h with brefeldin A (BFA, 2 µg/ml) or left untreated as negative control. B. Macrophages were pre-incubated for 30 min with BFA (2 µg/ml) or left untreated as negative control and further stimulated for 16 h with live *T. borreli* parasites (0.5×10^6^ per well). Percentage (%) of cells with an MFI higher than 10° was defined as % SITR^+^ cells for all the density plots based on the plot obtained with the negative isotype control. The density plots of intracellular SITR protein expression analysed by flow cytometry using anti-SITR antibody are representative of four experiments. Mean fluorescent intensity (MFI) of FITC and PE are represented in X and Y axes, respectively. C. Percentage SITR^high^ cell populations (averages ± SD of *n* = 4 fish) after pre-incubation for 30 min with BFA, or left untreated as negative control, followed by stimulation with live *T. borreli* parasites for 16 h. Symbol (*) indicates a significant (*P*≤0.05) difference in parasite stimulated cells compared with unstimulated cells.

To assess whether the observed decrease of intracellular staining for SITR upon stimulation with live *T. borreli* should be ascribed to secretion of SITR protein, an optimized concentration of brefeldin A (BFA) was used. BFA is an inducer of retrograde protein transport from the Golgi to the ER, leading to protein accumulation in the ER and therefore impairs protein secretion. The percentage of SITR+ macrophages reduced from 65% to 45%, approximately, after incubation with only BFA (Fig. 6A, C). Stimulation of macrophages with both BFA and live *T. borreli* parasites increased intracellular SITR protein expression from 55% to 70%, approximately (Fig. 6B, C). The use of an inhibitor of endosomal acidification (chloroquine) did not impair the secretion of SITR protein upon parasite stimulation, confirming the targeting of the protein to the extracellular space and not to the lysosomal compartment (data not shown). Hence, our results strongly suggest that stimulation of macrophages with live protozoan parasites promotes the secretion of SITR protein *in vitro*.

### Carp SITR is detected in the supernatant of parasite-stimulated cells *in vitro*

**Figure 7:**
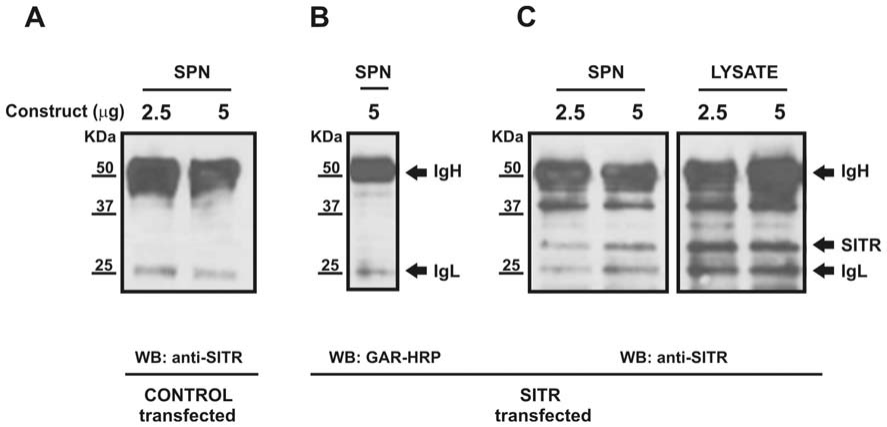
Detection of SITR protein in the supernatant of SITR-transfected HEK 293 cells. TLR2ÄTIR (negative control)- and SITR-transfected HEK 293 cells were stimulated with live *T. borreli* parasites (10^6^ per well) for 16 h. Supernatants and cell lysates from transfected HEK 293 cells were collected and SITR was immunoprecipitated using the affinity purified anti-SITR antibody. A. Western blots of immunoprecipitated supernatants (SPN) of TLR2ÄTIR-transfected (negative control, 2.5 or 5µg construct ) HEK 293 cells. SITR expression was evaluated using anti-SITR antibody. B. Western blots of immunoprecipitated supernatants (SPN) of SITR-transfected (5µg construct) HEK 293 cells. Co-elution of the heavy (IgH) and light chain (IgL) of the anti-SITR antibody during the immunoprecipitation protocol was confirmed using goat-anti-rabbit conjugated with horseradish peroxidase (GAR-HRP). C. Western blots of immunoprecipitated supernatants and cell lysates of SITR-transfected (2.5 or 5µg construct) HEK 293 cells. SITR expression was evaluated using anti-SITR antibody.

Sequence analysis classified carp SITR as a type I soluble protein expected to be secreted extracellularly. Furthermore, decrease of intracellular SITR protein expression after stimulation with *T. borreli* parasites and increase of intracellular SITR protein expression after co-stimulation with BFA suggested a parasite-induced secretion of SITR protein to the extracellular space. To validate secretion, SITR was immunoprecipitated in the supernatants of SITR-transfected HEK 293 cells stimulated with parasites. In supernatants of control-transfected HEK 293 cells, only the heavy (∼50KDa) and light chains (∼25KDa) of the anti-SITR antibody were detected (Fig. 7A). To confirm the co-elution of the IgH and IgL chains during the immunoprecipitation procedure, GAR-HRP was used as the only detecting antibody (Fig. 7B). Two additional bands were detected in western blots of both cell lysates and supernatant using anti-SITR antibody as detecting antibody, of which one with predicted MW for carp SITR of 28 KDa (Fig. 7A). The presence of SITR protein in supernatants of SITR-transfected cells only, confirms secretion of SITR protein to the extracellular space.

### Carp SITR may be secreted during *in vivo* infection with the protozoan parasite *T. borreli*

**Figure 8:**
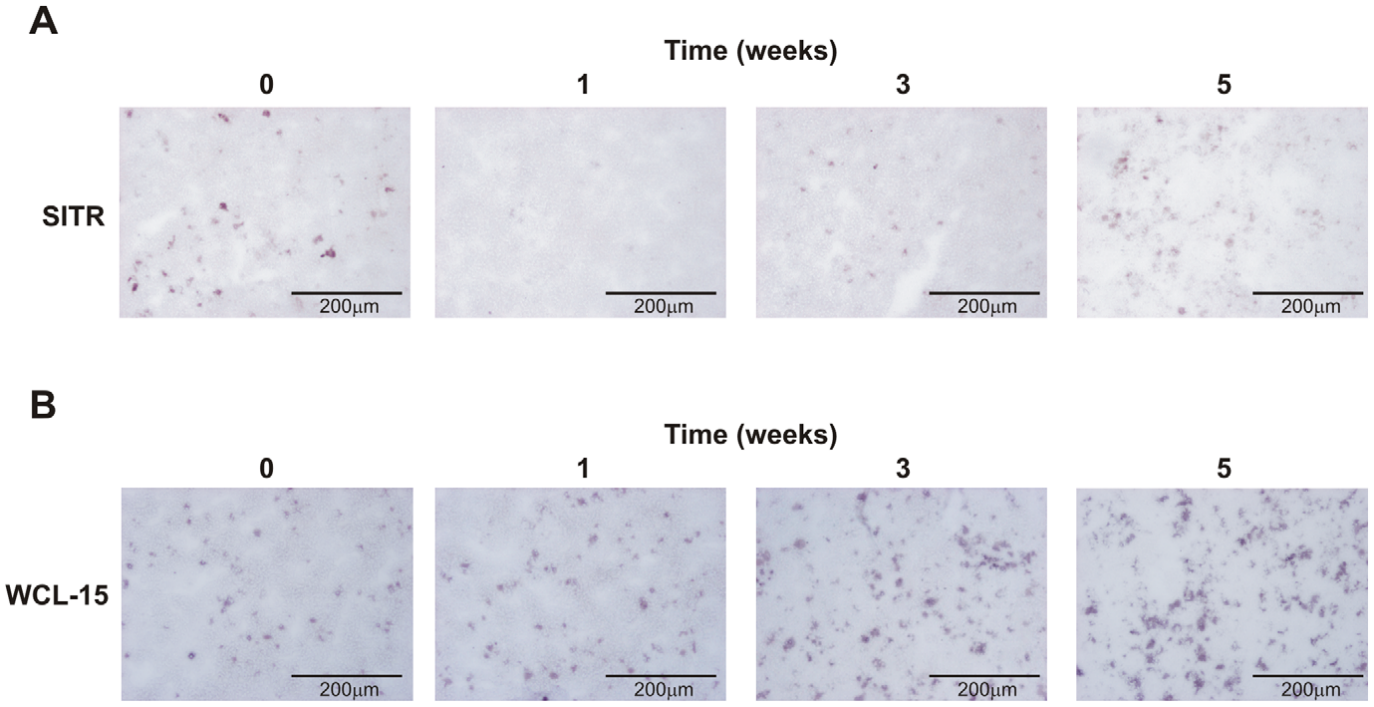
SITR protein expression during *in vivo T. borreli* infection. A. Anti-SITR immunoreactivity (blue) in spleen tissue from non-infected fish (control) and at 1, 3 and 5 weeks post-infection with *T. borreli* parasites. B. Staining for monocytes/macrophages (WCL-15; red) in spleen tissue from non-infected fish (control) and at 1, 3 and 5 weeks post-infection with *T. borreli* parasites.

*In vitro* stimulation of carp macrophages with *T. borreli* resulted in a decrease of intracellular staining indicative of SITR secretion. Immunohistochemical analysis of spleen of *T. borreli*-infected carp showed a similar decrease of SITR protein expression at week 1–3 post-infection, increasing to basal levels at 5 weeks post-infection (Fig. 8A). The simultaneous (moderate) increase rather than decrease in number of (WCL-15+) monocytes/macrophages (Fig. 8B), proved that the decrease in SITR protein expression during infection was independent of the absolute number of macrophages present. These results suggest a *T. borreli*-induced secretion of SITR protein also *in vivo*.

### Overexpression of carp SITR in RAW cells increases NO production

**Figure 9:**
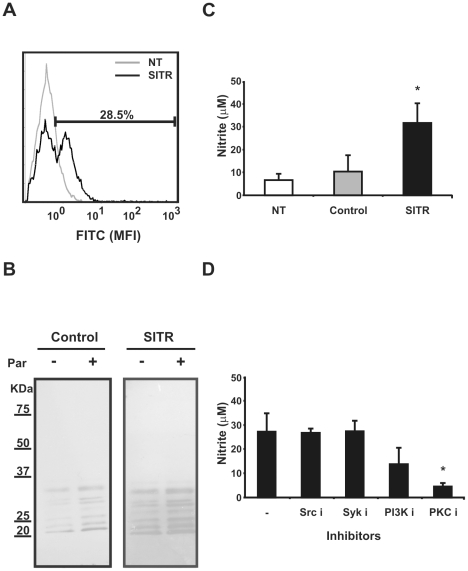
Overexpression of SITR in mouse RAW macrophages. A. Intracellular SITR protein expression in RAW cells analysed by flow cytometry using anti-SITR antibody (1∶50). RAW cells were non-transfected (NT) or transfected with carp SITR. B. Western blot of cell lysates of TLR2ΔTIR- (control) and SITR-transfected RAW cells stimulated with live *T. borreli* parasites (Par, 0.5×10^6^ per well) for 15 min or left untreated as control. Tyrosine phosphorylation was evaluated using an anti-phospho tyrosine antibody. C. Nitrite concentration (averages ± SD of *n* = 5) in supernatants of non-transfected (NT), TLR2ÄTIR-(control) and SITR- transfected RAW cells determined by Griess reaction at 24 h. Symbol (*) indicates a significant (*P*≤0.05) difference compared with TLR2ÄTIR - transfected RAW cells. D. Nitrite concentration (averages ± SD of *n* = 3) in supernatants of SITR- transfected RAW cells pre-incubated for 30 min with inhibitors of Src kinase (Src i, PP2, 20µM), Syk kinase (Syk i, Piceatannol, 50µM), and PI3K kinase (PI3K i, LY294002, 50µM), PKC kinase (PKC i, Staurosporine, 1µM) or left untreated as control. TLR2ÄTIR-(control)- transfected RAW cells were used as negative controls (data not shown). Nitrite levels were determined by Griess reaction at 24h. Symbol (*) indicates a significant (*P*≤0.05) difference compared with unstimulated cells.

To investigate macrophage functions associated with SITR activation, we transfected mouse (RAW) macrophages with a construct expressing the carp SITR gene. Transfection efficiency ranged between 25% and 35% as measured by intracellular staining using anti-SITR antibody (Fig. 9A). RAW cells transfected with carp TLR2 truncated at the TIR domain (TLR2ΔTIR), or non-transfected RAW cells, were used as negative controls.

The SITR protein has several predicted tyrosine phosphorylation sites as well as a protein kinase C (PKC) phosphorylation site (see Fig. 1A), suggesting that it could be involved in tyrosine phosphorylation- and PKC-dependent mechanisms. Total tyrosine phosphorylation was analysed by western blot using an anti-phospho tyrosine antibody. Stimulation of SITR-transfected RAW cells with *T. borreli* parasites resulted in an induction of tyrosine phosphorylation stronger than in negative controls (Fig. 9B). Nitrite production is one of the signature features of *T. borreli* infections in carp and is kinase dependent [37], [38]. We observed an increase of basal NO levels in SITR-transfected RAW cells, but not in negative controls (Fig. 9C). Inhibition of Src and Syk kinases did not inhibit SITR-induced NO production in transfected RAW cells. In contrast, inhibition of PKC kinase, and to some extent inhibition of PI3K kinase, resulted in an abrogation of SITR-induced NO production (Fig. 9D). These results suggest that *T. borreli*-induced stimulation of SITR results in the activation of tyrosine phosphorylation dependent cascades, including a PKC-dependent route that leads to NO production.

### Knock-down of SITR in carp macrophages down-regulates gene expression of iNOS

**Figure 10:**
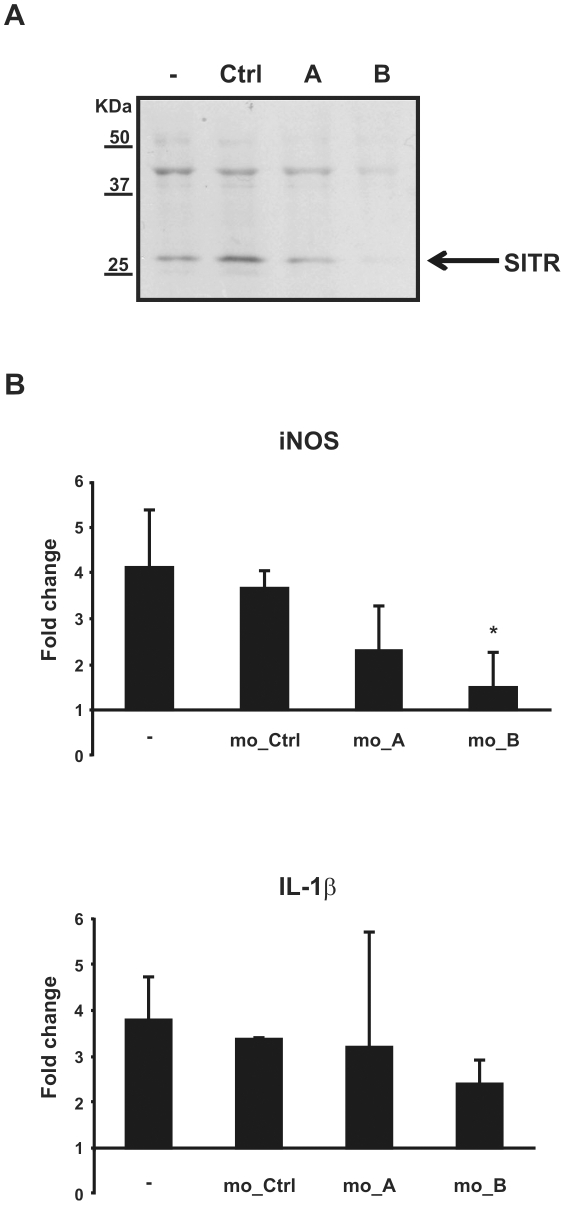
Knock-down of SITR protein in carp macrophages. A. Western blot of cell lysates from carp macrophages incubated for 48 h with control morpholino (control, 5µM), SITR morpholino A (SITR_A, 5 µM) or SITR morpholino B (SITR B, 5 µM) or left untreated as control. SITR protein expression was analysed using anti-SITR antibody. B. Real-time gene expression in carp macrophages pre-incubated for 48 h with control morpholino (mo_ctrl, 5 µM), SITR morpholino A (mo_A, 5 µM) or SITR morpholino B (mo_B, 5 µM) or left untreated. Macrophages were further stimulated for 6 h with live *T. borreli* parasites (0.5×10^6^) or left unstimulated. mRNA levels of inducible nitric oxide synthase (iNOS) and Interleukin-1β (IL-1β) are shown relative to the house keeping gene 40S ribosomal protein S11 and are expressed as fold change relative to unstimulated cells (fold change = 1). Bars show averages ± SD of *n* = 4 fish. Symbol (*) shows a significant (*P*≤0.05) difference compared to macrophages incubated with control morpholino.

Overexpression of carp SITR in RAW cells resulted in increased NO levels, suggesting a role for the SITR molecule in NO production. To verify SITR involvement in NO induction, antisense morpholinos were designed to knock-down SITR gene translation in carp macrophages. Use of a non-specific morpholino fused to 3′-carboxyfluorescein showed morpholino delivery was successful after 24 h, but maximal after 48 h. Successful inhibition of SITR translation was evaluated by intracellular staining and western blot with SITR antibody. Morpholino B, but not morpholino A nor the non-specific morpholino reduced the Mean Fluorescence Intensity (MFI) of SITR-positive macrophages (data not shown). Similarly, only use of morpholino B reduced the intensity of the 28 KDa SITR-specific band in western blot (Fig. 10A). Therefore, morpholino B was used to verify SITR involvement in NO induction by carp macrophages.

Stimulation of carp macrophages with live *T. borreli* parasites up-regulated gene expression of IL-1β and iNOS 5-fold approximately. Pre-incubation of carp macrophages with morpholino B, but not with non-specific morpholino, specifically reduced iNOS gene expression (Fig. 10B). These data show the involvement of SITR in protozoan parasite *T. borreli*-induced iNOS gene expression.

## Discussion

In this study, we describe the molecular cloning and functional characterization of a soluble immunoglobulin-like receptor SITR in teleost fish. Carp SITR has two extracellular Ig-domains with an unique organization: a V/C2 (or I-) type N-proximal Ig domain and a V-type C-proximal Ig domain. The carp SITR V-type Ig domain, in particular, has a close sequence similarity and conserved structural characteristics to mammalian CD300-like molecules. In contrast to the majority of IgSF receptors, SITR has no transmembrane domain. In carp, SITR is secreted by macrophages upon stimulation with protozoan parasites. Overexpression of SITR in mouse macrophages and knock-down of SITR in carp macrophages provides evidence for involvement of SITR in parasite-induced NO production.

Sequence analysis predicted carp SITR to have a N-proximal Ig-like domain of the V/C2- (or I-) type and a C-proximal Ig-like domain of the V-type. In general, Ig folds are formed by antiparallel β-strands arranged into two β-sheets linked by disulphide bonds and IgSF domains can be classified in V, C1, C2 and I types according to sequence patterns and length [3], [4]. By convention, the β-strands have been labeled *A* to *G* (based on the C1 domain) with the two additional strands in V–type Ig domains, present between *C* and *D*, labeled *C*′and *C*″. One β-sheet consists of β-strands *A*, *B*, *E* and possibly C′ and *C*″ while the other β-sheet contains strands *C*, *F*, *G*. Bork *et al.* [3] classified Ig-domains as belonging to V-types having all 9 β-strands, C1 types lacking the *C*′ and *C*″, C2 types having the *C*′ strand but not the *C*″ or *D* strands and I types, which are a hybrid between V- and C2-type Ig domains. In contrast to the C-proximal V-type Ig domain, unambiguous assignment of the N-proximal Ig domain of carp SITR as of the V/C2- (or I-) type remains challenging. If our analysis is correct, the organization of the carp SITR into an N-proximal Ig-like domain of the V/C2- (or I-) type and a C-proximal Ig-like domain of the V-type is unique [5], [6].

We used the SITR cDNA sequence to search for orthologues in the genome of zebrafish, a close relative of common carp [36]. This search identified 6 sites on 4 different chromosomes (1, 2a, 2b, 15a, 15b and 19) coding for SITR-related molecules of which several with multiple genes. Carp SITR and SITR-related molecules in zebrafish form a cluster of receptors that all appear to be soluble. However, it is difficult to reliably predict transmembrane exons from genomic sequences only and thus this prediction will require further research on cDNA sequences from zebrafish. In any case, these results suggest carp SITR to be an orthologue of a multigenic family of SITRs in zebrafish.

IgSF structural domain types differ in species distribution. V-types are found throughout all animal species in evolution. C1-types are found only in vertebrates and therefore must have evolved late during metazoan evolution. C2-type Ig domains have been described for *Drosophila melanogaster* and thus are considered to have originated in the protostome lineage. Presently, I-type (and V-type) domains have been found in sub-vertebrate species such as the sea anemone [39]. In general, about one-third of the characterized surface proteins of human leukocytes belong to the IgSF. Approximately half of these IgSF proteins contain two Ig domains; an N-proximal V-type followed by a C-proximal C2-type Ig domain [5]. In teleost fish, this type of organization can be found in, for example, the NITR family [32], [40]. The observed SITR Ig domain organisation may be unusual but is not unique to carp and seems a conserved feature of the SITR-related genes found in the zebrafish genome (unpublished data). The presence of this novel type of IgSF organization may be the result of exon shuffling through intronic recombination as has been described for other members of the IgSF [41], [42]. Exon shuffling by which domains can be inserted into a protein, or alternative splicing by which domains can be excluded from a protein, can contribute to addition and deletion of Ig domains from the middle of proteins and therefore give rise to a variety of organisations for IgSF proteins [43]. In conclusion, we have identified a protein that may stand as a model for a novel family of soluble immune-type receptors in fish that displays two unique structural features; (i) the Ig domains are organised as an N-proximal V/C2- (or I) type and a C-proximal V-type and (ii) SITR genes may represent genome-encoded soluble receptors.

The unambiguous assignment of carp SITR sequences as orthologs or paralogs of other presently known immune receptors is difficult at this point. Comparison of structure and chromosomal location of SITRs with previously described Ig-type receptors in zebrafish such as NITRs [31] clearly demonstrates that SITRs form distinct cluster(s) of immune receptor genes. Although alignment and homology searches show a close resemblance of the C-proximal V-type Ig domain of SITRs with human CD300 genes, members of the CD300 family have a single (V-type) Ig domain and usually are transmembrane rather than soluble receptors. CD300 molecules comprise a family of seven members. All members, except for CD300G, possess structural motifs with stimulatory or inhibitory potential [18]. Cross-linking CD300 molecules on different leukocyte populations broadly affects gene transcription, phagocytosis, cytokine production, migration and survival [44], [45], [46]. The cell surface expression of CD300 family members is modulated, in part, by the ability of these molecules to internalize whereas effective CD300 signalling appears to be induced by clustering e.g. into lipid rafts [47]. Likely, following the confirmation of carp SITR as a member of a larger family of soluble CD300-related molecules in teleost fish, continued studies on SITRs should provide further insight into the evolution of the CD300 family of molecules.

As predicted from the sequence analysis, SITR protein is not found in the cell membrane but located intracellularly in vesicle-like structures. Upon *in vitro* stimulation of macrophages with live *T. borreli* parasites SITR protein is readily secreted within minutes. Likewise, we observed a secretion of SITR protein *in vivo* in spleen of *T. borreli-*infected fish already at week 1 post-infection. The detection of SITR protein in supernatants of parasite-stimulated SITR-transfected cells corroborated the secretion, rather than degradation, of carp SITR to the extracellular space.

To assess the function of SITR, we increased SITR protein expression by overexpression in mouse RAW macrophages or reduced SITR protein expression by knock-down in carp macrophages. Cellular activation upon overexpression or inhibition of SITR protein expression was evaluated by means of radical production, phosphorylation analysis and gene expression. Carp SITR itself could act as receptor when overexpressed in mouse macrophage RAW cells and also human HEK cells (unpublished data). Stimulation of these SITR transfectants with live *T. borreli* parasites promoted tyrosine phosphorylation-dependent intracellular signaling cascades. Also, overexpression of SITR in RAW cells increased NO production. The NO induction appeared to be PKC- and partly PI3K-dependent, corroborating the predicted ability of SITR to interact with the PKC kinase and revealing the potential of SITR activation to initiate a phosphorylation-dependent signaling cascade upon stimulation with protozoan parasites. The ability of IgSF receptors to associate with kinases has been proven to exist already in the earliest metazoans such as poriferans [48]. Thus, carp SITR (Ig-like domains) proteins may have retained this function. Nitrite production is one of the signature features of *T. borreli* infections of carp as has been shown by strongly increased iNOS gene expression in head-kidney and spleen, increased serum nitrite levels and extensive tyrosine nitration in the spleen [37], [49]. *Ex vivo* restimulation of macrophages from *T. borreli*-infected carp with LPS or parasite lysates indicated the presence of classically activated macrophages (caMF) during *T. borreli* infection [50]. Furthermore, NO production during *T. borreli* infection was shown to be protein tyrosine kinase (PTK) and PKC-dependent [38]. However, despite all the information acquired on the NO production induced by *T. borreli*, the innate immune receptors implicated in this production are still largely unknown.

Certainly, the increased SITR gene expression in *T. borreli*-stimulated macrophages and the secretion of SITR protein during *in vivo T. borreli* infection suggests a role for this IgSF receptor in the host response to this protozoan parasite. Knock-down of SITR protein expression in carp macrophages, using morpholino antisense technology, confirmed the involvement of carp SITR in the induction of iNOS gene expression by *T. borreli*. A hypothesis could be that an intracellular activating signal from a cell surface-bound receptor, upon recognition of parasite-derived ligand, promotes the interaction of PKC kinase with intracellular SITR and initiate a phosphorylation-dependent cascade leading to NO production. In this situation, extracellular secretion of carp SITR could either represent a strategy to counter-regulate the concentration of intracellular SITR or a strategy to facilitate/antagonize the intracellular SITR-dependent activation [23], [51].

Collectively, this study provides a comprehensive analysis, not only *in vitro* but also *in vivo*, of the regulation and putative biological activity of SITR in carp. Further molecular and functional characterization of additional members of an apparent larger family of SITR genes will shed light on the specificity and complementarity of the mechanisms of action between SITR receptors and other innate immune receptors. Finally, we propose a role for carp SITR in the NO-mediated response to the protozoan parasite *Trypanoplasma borreli*.

## Materials and Methods

### Ethics statement

All animals were handled in strict accordance with good animal practice as defined by the relevant national and/or local animal welfare bodies, and all animal work was approved by the animal experimental committee of Wageningen University, Wageningen, The Netherlands. (license numbers: 2004079/2004137/2008054).

### Animals

European common carp (*Cyprinus carpio carpio* L.) were reared in the central fish facility of Wageningen University, The Netherlands at 23°C in recirculating UV-treated tap water and fed pelleted dry food (Skretting, Nutreco) daily. R3×R8 carp are the hybrid offspring of a cross between fish of Hungarian origin (R8 strain) and of Polish origin (R3 strain) [52]. Carp were between 9 and 11 months old at the start of the experiments.

### Parasites

*Trypanoplasma borreli* was cloned and characterized by *Steinhagen et al.* [53]. Parasites were maintained by syringe passage through carp. Parasitaemia was monitored in 10× diluted blood in cRPMI [RPMI 1640 (Invitrogen, CA, USA) adjusted to carp osmolarity 280 mOsmkg^−1^ containing 50 U/ml of heparin (Leo Pharma BV, Weesp, The Netherlands)] using a Bürker counting chamber. The minimum detection limit by this method was 10^5^ parasites/ml of blood (viability>95%). For parasite isolation, blood was collected from 3-weeks-infected fish and purified on a 1×12cm ion-exchange chromatography using DEAE cellulose (DE-52; Whatman international) [54]. After purification, parasites were resuspended in HML medium [55] supplemented with 5% pooled carp serum, L-glutamine (2 mM, Cambrex, Verviers, Belgium), penicillin G (100 U/ml, Sigma-Aldrich), and streptomycin sulfate (50 mg/l, Sigma-Aldrich).

### Reagents

Inducer of retrograde protein transport Brefeldin A (BFA) from *Penicillium brefeldianum* and LPS from *Escherichia coli* were purchased from Sigma-Aldrich (St. Louis, MO). Inhibitor of endosomal acidification chloroquine and inhibitor of phosphatidylinositol 3-kinase (PI3K) LY294002 were purchased from InvivoGen (Cayla SAS, France). Syk tyrosine kinase inhibitor Piceatannol was purchased from Bio-connect (Tocris Biosciences, Missouri, USA) and Src tyrosine kinase inhibitor 4-amino-5-(4-chlorophenyl)-7-(t-butyl)pyrazolo[3,4-d]pyrimidine (PP2) was purchased from Gibco (Invitrogen, CA, USA). Protein kinase C (PKC) inhibitor Staurosporine was purchased from Alexis Biochemicals (San Diego, CA, USA).

### Generation of a subtracted cDNA library

A substracted cDNA repertoire was generated by Suppression Substraction Hybridization (SSH) using the PCR (polymerase chain reaction)-Select cDNA substraction kit Catalog no. 637401 (Clontech, Palo Alto, CA). cDNA from macrophages stimulated with live *T. borreli* parasites for 6h was used as tester and cDNA from unstimulated macrophages was used as a driver. Tester and driver samples represent a pool of cDNA samples from n = 4 fish. The substracted cDNA repertoire was amplified by PCR according to the manufacturers protocol of the PCR-Select cDNA substraction kit (Clontech). The resulting PCR products were ligated and cloned into JM-109 cells using pGEM-Teasy kit (Promega) according to the manufacturer's protocol. Two hundred and eighty-eight clones were picked and sequenced. A nucleic-acid homology search revealed that out of 288 clones, 3 clones represented the SITR molecule described in this manuscript.

### Amplification of carp SITR full-length cDNA

Oligonucleotide primers for carp Soluble Immune-Type Receptor (SITR) were designed based on the (partial) consensus sequence of 274bp obtained by SSH. cDNA from macrophages stimulated with *T. borreli* for 6h was used as template for PCR or nested PCR. The 5′and 3′ends of SITR were amplified using gene-specific primers (Forward SITR: ATTTCAGTCGGATTTTGGCTCAG and Reverse SITR: CGTAGCTTTCAACACCTAAACTGAGC) by 5′and 3′rapid amplification of cDNA ends (RACE) using the Gene Racer™ RACE Ready cDNA kit (Invitrogen, Breda, The Netherlands) according to the manufacturer's protocol. PCR reactions were performed in *Taq* buffer, using 1U *Taq* polymerase (Promega, Leiden, The Netherlands) supplemented with MgCl_2_ (1.5 mM), dNTPs (200 µM) and primers (400 nM each) in a total volume of 50 µl. PCR and nested PCR were carried out under the following conditions: one cycle 4 min at 96°C; followed by 35 cycles of 30 sec at 96°C, 30 sec at 55°C and 2 min at 72°C; and final extension for 7 min at 72°C, using a GeneAmp PCR system 9700 (PE Applied Biosystems, Foster City, CA). Products amplified by PCR, nested PCR or RACE-PCR were ligated and cloned in JM-109 cells using the pGEM-Teasy kit (Promega) according to the manufacturer's protocol. From each product both strands of eight clones were sequenced, using the ABI prismBigDye Terminator Cycle Sequencing Ready Reaction kit and analysed using 3730 DNA analyser.

### Bioinformatic analysis of carp SITR sequence

Nucleotide sequence was translated using the ExPASy translate tool (http://us.expasy.org/tools/dna.html) and aligned with Clustal W (http://www.ebi.ac.uk/clustalw). The signal peptide cleavage site and the transmembrane region was predicted by using the SignalP 3.0 (http://www.cbs.dtu.dk/services/SignalP/) and the TMHMM 2.0 (http://www.cbs.dtu.dk/services/TMHMM-2.0/) servers, respectively. Subcellular location was predicted using the TargetP (http://www.cbs.dtu.dk/services/TargetP/) server. Post-translational modifications were predicted using the NetNGlyc 1.0 (http://www.cbs.dtu.dk/services/NetNGlyc/), the NetPhos 2.0 (http://www.cbs.dtu.dk/services/NetPhos/) and the NetPhospho K1.0 (http://www.cbs.dtu.dk/services/NetPhosK/) servers. Homology searches were performed using blastp http://www.expasy.org/tools/blast/ and the WU-Blast http://www.proweb.org/Tools/WU-blast.html servers. Identification of protein domains were predicted using PFAM (http://pfam.sanger.ac.uk/) and SMART (http://smart.embl-heidelberg.de/) servers. Prediction of â-strands for each Immunoglobulin domain was predicted using Swissmodel (http://swissmodel.expasy.org/) and PSIPRED (http://bioinf.cs.ucl.ac.uk/psipred/) servers.

### Zebrafish SITR sequences retrieval and phylogenetic analysis

Chromosomes (Chr) within the zebrafish genome database were searched by basic local alignment search tool (BLAST) analysis [56] using the amino acid sequences for the carp SITR protein. Subsequently, the DNA surrounding homologues of this gene was retrieved (∼400,000 bp) for further analysis with the following sequence software programs; Genscan [57] identified possible coding regions within the genomic DNA, whereas the amino acid sequences were analysed using BLAST [56] and FASTA [58]. Phylogenetic relationships were constructed from ClustalX v1.81 [59] generated alignments of the full-length amino acid sequences of the known SITR-related molecules using the Neighbor-joining method [60]. The tree was drawn using TreeView v1.6.1 [61] and confidence limits added [62].

### Macrophage cell culture

Head kidney-derived macrophages, considered the fish equivalent of bone marrow-derived macrophages, were prepared as previously described [63], [64]. Briefly, carp head-kidneys were gently passed through a 100 µm sterile nylon mesh (BD Biosciences, Breda, The Netherlands) and rinsed with homogenization buffer [incomplete-NMGFL15 medium containing 50 U/ml penicillin G, 50 µg/ml streptomycin sulphate, and 20 U/ml heparin (Leo Pharmaceutical, Weesp, The Netherlands)]. Cell suspensions were layered on 51% (1,07 g.cm^−3^) Percoll (Amersham Biosciences, Uppsala, Sweden) and centrifuged at 450 *g* for 25 min at 4°C with the brake disengaged. Cells at the interphase were removed and washed twice in incomplete NMGFL-15 medium. Cell cultures were initiated by seeding 1.75×10^7^ (viability>95%) head kidney leukocytes in a 75 cm^2^ culture flask containing 20 ml of complete NMGFL-15 medium [incomplete-NMGFL-15 medium supplemented with 5% heat-inactivated pooled carp serum and 10% fetal bovine serum]. Head kidney-derived macrophages, named macrophages throughout the manuscript, were harvested after 6 days of incubation at 27°C by placing the flasks on ice for 10 min prior to gentle scraping.

### Gene expression analysis

Total RNA was isolated using the RNeasy Mini Kit (Qiagen, Leusden, The Netherlands) including the accompanying DNase I treatment on the columns, according to the manufacturers' protocol and stored at −80°C until further use. Prior to cDNA synthesis, a second DN*ase* treatment was performed using DN*ase* I, Amplification Grade (Invitrogen). Synthesis of cDNA was performed with Invitrogen's SuperScript™ III First Strand Synthesis Systems for RT-PCR using random primers according to the manufacturer's instructions. A non-reverse transcriptase control was included for each sample. cDNA samples were further diluted 50 times in nuclease-free water before use as template in real-time PCR experiments. Real time quantitative PCR (RT-qPCR) was performed in a 72-well Rotor-Gene™ 6000 (Corbett Research, Mortlake, Sydney, Australia) with the Brilliant® SYBR® Green QPCR (Stratagene, La Jolla, CA, USA) as detection chemistry as previously described [64]. The primers used for RT-qPCR are listed in Table 1. Fluorescence data from RT-qPCR experiments were analysed using Rotor-Gene version 6.0.21 software and exported to Microsoft Excel. The cycle threshold *C*_t_ for each sample and the reaction efficiencies (*E*) for each primer set were obtained upon Comparative Quantitation Analysis from the Rotor-Gene version 6.0.21 software. The relative expression ratio (*R*) of a target gene was calculated based on the *E* and the *C*_t_ deviation of sample versus control [65], [66], and expressed relative to the S11 protein of the 40S subunit as reference gene.

**Table 1:** Primers used for real-time qPCR analysis.

Primer	Sequence (5′-3′)	GenBank Accession No.
40S Fw	CCGTGGGTGACATCGTTACA	AB012087
40S Rv	TCAGGACATTGAACCTCACTGTCT	
TLR2 Fw	TCAACA+CTCTTAATG+TGAGCCA a	FJ858800
TLR2 Rv	TGTG+CTGGAAA+GGTTCAGAAA a	
SITR Fw	GCTCCTGATGTGT+CTGTGGTGA a	HM370297
SITR Rv	CTCC+CCACTGTG+TAACAGC a	
iNOS Fw	AACAGGTCTGAAAGGGAATCCA	AJ242906
iNOS Rv	CATTATCTCTCATGTCCAGAGTCTCTTCT	
IL-1â Fw	AAGGAGGCCAGTGGCTCTGT	AJ245635
IL-1â Rv	CCTGAAGAAGAGGAGGAGGCTGTCA	

a The ‘+’ is before the nucleic acid in which the locked nucleic acid was placed.

### Morpholino delivery in carp macrophages

A morpholino knockdown approach was used to knock-down SITR protein expression in carp macrophages by inhibition of SITR mRNA translation according to the manufacturer's instructions (Gene Tools, LLC, Philomath, USA). Two antisense morpholino (Gene Tools) were designed: SITR_morpholino_A (mo_A) and SITR_morpholino_B (mo_B). mo_A (5′-TCTTCGTGTAGGAGGCCATTTCTTT-3′) targets the carp SITR mRNA at positions −6 to +19 with respect to the ATG. mo_B (5′-CTCTTTGCTGATGTTTCCTGTAAGA-3′) targets the carp SITR mRNA at positions -32 to -7 with respect to the ATG. As a negative control, we used a standard control (mo_ctrl, 5′-CCTCTTACCTCAGTTACAATTTATA-3′) which was expected to have no target and no biological activity in carp macrophages (Gene Tools). The standard control was fused to carboxyfluorescein to estimate the efficiency of morpholino delivery. Carp macrophages were resuspended in rich-NMGFL-15 medium [incomplete-NMGFL-15 medium supplemented with 2.5% heat-inactivated pooled carp serum and 5% fetal bovine serum] and mo_control (5 µM), mo_A (5 µM), mo_B (5 µM) and Endo-Porter (6 µM) were added. The mix was immediately swirled, and after 48h of incubation, the efficiency of morpholino activity was tested by means of intracellular staining and western blot analysis of protein expression and by gene expression analysis.

### Primary Antibodies

Mouse monoclonal antibody WCI-15 strongly reacts with the cytoplasm of carp monocytes and macrophages in tissue sections [67]. Mouse monoclonal antibody TCL-BE8 binds to carp neutrophilic granulocytes (strong affinity), monocytes (low affinity) [68]. Mouse monoclonal antibody WCI-12 binds to the heavy chain of IgM in carp B cells [69], [70]. Mouse monoclonal antibody WCL6 recognizes a 90KDa membrane molecule on carp thrombocytes [71]. Mouse monoclonal anti-phosphotyrosine antibody and rabbit IgG anti-β-tubulin antibody were purchased from Abcam (Cambridge, UK).

Polyclonal rabbit antibodies anti-carp SITR were produced against each of two synthetic peptides coupled to keyhole limpet hemocyanin (KLH), according to a 3-months standard protocol (Eurogentec S.A., Seraing, Belgium). For peptide 1, amino acids 45-60 (CYYDKKYTQQKKYWYS) and for peptide 2, amino acids 169–185 (YTVGRTDTSQNSSVQIS) of the carp SITR protein were chosen for immunization. Each of the peptide is present on a different Ig domain of the SITR protein. Affinity purification of rabbit IgG was performed against purified peptides and specificity assessed by ELISA (Eurogentec). Anti-SITR antibody produced against peptide 1 binds to a 28KDa protein as assessed by western blot analysis but, in contrast to anti-SITR produced against peptide 2, does not lead to any positivity when assessed by flow cytometer or immunohistochemistry. For this reason we measured SITR protein expression using anti-SITR antibody produced against peptide 2 and used the remaining anti-SITR antibody as isotype control.

### Magnetic activated cell sorting (MACS)

Macrophage-enriched fractions of head kidney leukocytes were obtained essentially as previously described [64]. Cell suspensions were layered on a discontinous Percoll gradient (1.020, 1.060, 1.070 and 1.083 g cm^−3^) and centrifuged 30 min at 800 *g* with the brake disengaged. Cells at 1.070 and 1.083 g cm^−3^ were collected, pooled and washed twice with cRPMI [RPMI 1640 adjusted to carp osmolarity 280 mOsmkg^−1^]. The monoclonal antibody TCL-BE8 (1∶50) was used to separate neutrophilic granulocytes from macrophages by MACS as previously described. Briefly, after incubation for 30 min with TCL-BE8 on ice, the leukocyte suspension was washed twice with cRPMI and incubated with phycoerythrin (PE)-conjugated goat anti-mouse (1∶50; DAKO, Glostrup, Denmark) for 30 min on ice. The magnetic separation was performed on LS-MidiMACS Columns (Mitenyi Biotec) according to the manufacturer's instructions. The purity of the TCL-BE8^+^ (neutrophilic granulocyte-enriched fraction; >90%) and TCL-BE8^−^ (macrophage-enriched fraction; <10%) fractions was confirmed by flow cytometric analysis using a FACScan® flow cytometer (Becton Dickinson, Mountain View, CA, USA).

To obtain B cell-, T cell- and thrombocyte-enriched fractions, blood was collected by puncture of the carp caudal vessel using a heparinised (Leo Pharmaceuticals Products Ltd, Weesp, The Netherlands) syringe. Blood was centrifuged 15 min at 800 *g* at 4°C. The buffy coat was collected and layered on 3 ml Ficoll (density 1.077 g cm^−3^, Amersham Biosciences, Uppsala, Sweden). Following subsequent centrifugation at 800 *g* at 4°C for 25 min with the brake disengaged, cells at the interface were collected and washed twice with cRPMI. The monoclonal antibody WCI-12 (1∶50) was used to separate B-cells and the monoclonal antibody WCL-6 (1∶50) was used to separate thrombocytes by MACS as above described. The purity of the WCI-12^+^ (B cell-enriched; >95%), WCI-12^−^ (T cell-enriched) and WCL-6^+^ (thrombocyte-enriched; >95%) fractions were confirmed by flow cytometric analysis using a FACScan® flow cytometer (Beckman Coulter, Epics XL-MCL, Miami, Florida, USA).

### Western blot analysis

Carp macrophages or mouse RAW macrophages were resuspended by pippeting and transferred to pre-cooled eppendorf tubes. Cells were washed twice in ice-cold PBS, lysed on ice with lysis solution [0.5% Triton X-100, 20 mM Tris, 100 mM NaCl, 1 mM EDTA, 50 mM NaF (Sigma), 5µM Na_3_VO_4_ (Sigma) 1 mM phenylmethylsulfonyl fluoride (PMSF, Sigma)], homogenized with a syringe and incubated 10 min on ice. Cell lysates were centrifuged at 21000 *g* for 10 min at 4°C. Supernatant was collected and total protein content was determined by the Bradford method. Samples (20–25 µg) were boiled at 96°C for 10 min with loading buffer containing â-mercaptoethanol and separated by 10% or 12.5% SDS-PAGE and electrophoretically transferred to nitrocellulose membranes (Protrans, Schleicher & Schuell, Bioscience GmbH). Membranes were blocked in 5% w/v milk powder in TBS-T (10 mM Tris, 150 mM NaCl, 0.1% Tween-20, pH 7.5) for 1 h at room temperature and then incubated with primary antibody overnight at 4°C in 5% w/v BSA in TBS-T. Following antibodies were used: polyclonal rabbit anti-SITR antibody (1∶100), mouse monoclonal anti-phosphotyrosine antibody (1∶500) and rabbit IgG anti-â-tubulin (1∶500). Membranes were then incubated with goat-anti-mouse HRP-conjugated (1∶1000, Dako, Glostrup, Denmark) or goat-anti-rabbit HRP-conjugated (1∶2000, Dako) in 5% w/v milk powder in TBS-T for 1 h at room temperature. Between each incubation step, membranes were washed three times in TBS-T for 10 min at room temperature. Signal was detected by development with a chemoluminescence kit (Amersham) according to the manufacturer's protocol and visualized by the use of Lumi-film chemiluminescent Detection Film (Roche, Woerden, The Netherlands).

### Intracellular SITR staining

For intracellular staining of SITR in carp macrophages, 1×10^6^ cells were resuspended in 50µL FACS buffer (0.5% BSA, 0.01% in PBS) and transferred to a 96-well U-bottom plate. For intracellular staining of SITR in mouse RAW macrophages, 0.25×10^6^ cells were resuspended in 50µL FACS buffer and transferred to a 96-well U-bottom plate. Following steps were perfomed on ice unless stated otherwise. Cells were first incubated 20min with blocking solution (10% foetal bovine serum in PBS) to reduce non-specific immunofluorescent staining. After washing with FACS buffer, cells were permeabilized by incubation for 15min with 100µL Cytofix/Cytoperm (BD Bioscience, California, USA). After a washing step with 1× Perm/Wash buffer (BD Bioscience), cells were incubated for 30min with affinity-purified polyclonal rabbit anti-SITR antibody or with the isotype control (both at 1∶10 in Perm/Wash buffer). After washing 1× Perm/Wash buffer, cells were incubated for 30min in the dark with the swine-anti-rabbit antibody conjugated with fluorescein isothiocyanate (SWAR-FITC, 1∶50, Dako) as secondary antibody. After extensive washing with 1× Perm/Wash buffer, cells were transferred to flow cytometer tubes. Fluorescent intensities of 10^4^ events were acquired in log scale using a Beckman Coulter Epics XL-MCL flow cytometer. Incubation with the isotype control (anti-SITR antibody produced against peptide 1) lead to no positive reaction.

### Immunohistochemistry

Cryosections (7 µM) of spleen tissue were mounted on poly-*L*-lysine-coated glass slides (BDH Laboratory Supplies, Poole, UK), air-dried for 60 min and incubated in a 0.3% H_2_O_2_ solution in methanol for 20 min to inactivate endogenous peroxidase. Following steps were performed at room temperature unless stated otherwise. Cryosections were washed for 5 min with PBS, then short with distilled water and incubated in proteinase-K solution (50 µg/ml in distilled water) for 10 min at 37°C. Samples were fixed in 4% paraformaldehyde in PBS for 10 min at 4°C followed by washing in 0.1% Triton PBS (PBS-T) for 10 min at 4°C and subsequently in PBS-T for 7 min at room temperature. A blocking solution of 5% normal goat serum was then added onto the slides and incubated for 30min. Affinity-purified polyclonal rabbit anti-SITR antibody (1∶10) was then added alone or in combination with the mouse monoclonal antibodies WCL-15 (1∶50), TCL-BE8 (1∶50), WCI-12 (1∶50) or WCL-6 (1∶50) in PBS for 1h. After washing twice for 10min in PBS-T, sections were incubated with the secondary antibody for 1h with goat anti-rabbit antibody conjugated to alkaline-phosphatase (GAR-AP, Dako, 1∶150 in PBS) alone or in combination with goat anti-mouse antibody conjugated to horseradish peroxidase (GAM-HRP, Dako, 1∶150 in PBS). When only GAR-AP antibody was used, sections were first incubated in AP-buffer (0.1M Tris-Cl, 0.1M NaCl, 0.05M MgCl_2_, pH 9.5) for 10min and then stained using AP substrate [4.5 µl/ml nitro-blue-tetrazoleum (Roche Applied Science) and 3.5 µl/ml 5′-bromo-4′-chloro-3′-indolyl phosphatase (BCIP; Roche Apllied Science) in AP buffer] for 2–5 min followed by four washes in distilled water. Alternatively, when both secondary antibodies were used (double-staining), sections were first AP stained as described above. After rinsing four times with distilled water, sections were incubated for 10 min in 0.05 M sodium acetate buffer, pH 5 and following addition of 0.4 mg/ml 3-amino-9-ethyl-carbazole (AEC; Sigma-Aldrich) in sodium acetate buffer containing 0.03% H_2_O_2_ and incubated for 25 min. Finally, cryosections were rinsed four times in distilled water and embedded in Kaiser's glycerine gelatin (Merck, Darmstadt, Germany). As negative immunohistochemistry controls, cryosections were first incubated with the rabbit anti-SITR antibody followed by only GAM-AP or first incubated with mouse WCL-15, TCL-BE8, WCI-12, WCL-6 antobodies followed by only GAR-HRP. As positive immunohistochemistry controls, cryosections were first incubated with the rabbit anti-SITR antibody followed by GAR conjugated with HRP (GAR-HRP) or first incubated with mouse WCL-15, TCL-BE8, WCI-12, WCL-6 antibodies followed by GAM conjugated with AP (GAM-AP).

### Confocal laser scanning microscopy

Cytospins on poly-*L*-lysine coated glass slides (BDH Laboratory supplies) of TCL-BE8 negative fraction (MACS sorted) or carp macrophages were made by fixing in 100% alcohol and 99% acetic acid (10∶1). Mouse monoclonal WCL-15 (1∶50) antibody and /or polyclonal rabbit anti-SITR (1∶10) antibody were used as primary antibodies. Goat anti-mouse antibody conjugated to fluorescein isothiocyanate (GAM-FITC, Dako, 1∶50 in PBS) and goat anti-rabbit conjugated to tetramethylrhodamine-5-(and 6)-isothiocyanate (GAR-TRITC, Dako, 1∶50 in PBS) were used as secondary antibodies. Cytospins were embedded in Vectashield Mounting medium (Vector Laboratories) and examined with a Zeiss LSM-510 laser scanning microscope. FITC (green) signal was excited with a 488 nm argon laser and detected using a band-pass filter (505–530 nm) and TRITC (red) signal was excited with a 543 nm helium-neon laser and detected using a long-pass filter (560 nm).

### SITR-GFP and TLR2ΔTIR-GFP expression plasmids

The vivid color™pcDNA™6.2/C-EmGFP-GW/TOPO® (Invitrogen, catalog no. K359-20) expression vector combined with TOPO®cloning was used to fuse full-length SITR or TLR2ΔTIR (TLR2 truncated at TIR domain, [64] to EmGFP at the C-terminal end. Isolation of highly pure plasmid DNA suitable for transfection was performed using S.N.A.P.™ Midi Prep Kit (Invitrogen, catalog no. K1910-01) according to the manufacturer's protocol. C-terminal fluorescent-tagged protein could be visualized using confocal microscopy.

### Transient transfection of HEK 293 cells

Human embryonic kidney HEK 293 cells were cultured in DMEM supplemented with 10% FBS, 50 U/ml penicillin G and 50 µg/ml streptomycin sulphate. Two days prior to transfection, HEK 293 cells were seeded into tissue culture flasks to reach 80–90% confluence at the day of transfection. For transfection of HEK 293 cells, 2.5 or 5 µg of the carp SITR-GFP or TLR2ΔTIR-GFP (negative control) constructs was transfected into HEK 293 by nucleoporation using nucleofactor™ solution V and program A-23 (Lonza Cologne AG, Germany) according to the manufacturer's instructions. Forty-eight hours after transfection, cells were trypsinized (0.5% trypsin, GIBCO) and plated overnight in a 24-well plate, as described earlier [72]. The next day, cells were stimulated for 16 h with live *T. borreli* parasites (10^6^ per well). Cells and supernatants were collected and used for immunoprecipitation using affinity-purified polyclonal rabbit anti-SITR antibody and SITR expression was evaluated by western blot analysis.

### Immunoprecipitation protocol

Supernatants of TLR2ΔTIR-GFP (negative control)- or SITR-transfected HEK293 cells stimulated with live *T. borreli* parasites were centrifuged at 800 *g* for 10 min and the parasite pellet discarded. SITR-transfected HEK293 cells were lysed with ice-cold IP Lysis/Wash buffer (Pierce Biotechnology, Rockford, USA), the cell lysate centrifuged at 13000 *g* for 10 min at 4°C to pellet the cell debris and the remaining supernatant collected. Immunoprecipitation of SITR protein in these two preparations was performed using a Thermo Scientific Pierce Classic IP kit (catalog no. 26146, Pierce Biotechnology, Rockford, USA). Briefly, 20 µg of affinity purified anti-SITR antibody was combined with the two preparations and incubated overnight at 4°C to form immune complexes. Following centrifugation steps were performed at 1000 *g* for 1 min at 4°C. Then, 20 µl of the Protein A/G Agarose resin (Pierce Biotechnology, Rockford, USA) was added into a Pierce Spin Column, washed three times with ice-cold IP Lysis/Wash buffer and the flow-through discarded. The immune complexes were added to the resin in the column and the column was incubated with gentle end-over-end shaking for 1 h at 4°C. The resin was washed four times with 200 µl IP Lysis/ Wash buffer and once with 100 µl Conditioning Buffer (Pierce Biotechnology, Rockford, USA). Then, 50 µl of reducing Sample Buffer (Pierce Biotechnology, Rockford, USA) was added to the resin and incubated at 100°C for 10 min. Co-elution of antibody and immunoprecipated SITR was obtained after centrifugation and evaluated by western blot analysis.

### Transient transfection of murine RAW 264.7 macrophages

Mouse macrophage RAW 264.7 cell line was cultured in RPMI (Invitrogen, CA, USA) supplemented with 10% fetal bovine serum (FBS, Invitrogen) and 50 U/ml penicillin G (Sigma-Aldrich) and 50 µg/ml streptomycin sulphate (Sigma-Aldrich). One day before transfection, RAW cells were seeded into tissue culture flasks to reach 60–70% confluence at the day of transfection. For transient transfection, 2.5 µg of the carp SITR-GFP or TLR2ΔTIR-GFP constructs were transfected into RAW cells by nucleoporation using nucleofactor™ solution V and program D-32 (Lonza Cologne AG, Germany) according to the manufacturer's instructions. For western blot analysis, 24hours after transfection, cells were scraped, counted using Trypan blue exclusion and plated overnight at a concentration 5×10^5^ cells/ well in a 24-well tissue culture plate. The next day, cells were stimulated with 5×10^5^ live *Trypanoplasma borreli* parasites for 15 min or left untreated as control and lysed on ice with lysis solution [see Western blot section]. For measurement of NO production, 24hours after transfection, cells were scraped, counted using Trypan blue exclusion and plated overnight at a concentration of 2×10^5^ cells/ well in a 96-well tissue culture plate. The next day, 75 µl supernatant was collected and nitrite production was measured.

### Nitrite production

Nitrite production was measured essentially as described before [73]: to 75 µl of cell culture supernatant, 100 µl of 1% sulfanilamide in 2.5% (v/v) phosphoric acid and 100 µl of 0.1% (w/v) *N*-naphthyl-ethylenediamine in 2.5% (v/v) phosphoric acid were added in a 96-well flat-bottom plate. The absorbance was read at 540 nm (with 690 nm as a reference) and nitrite concentration (µM) was calculated by comparison with a sodium nitrite standard curve.

### Statistical Analysis

Transformed values (ln) were used for statistical analysis in SPSS software (version 17.0). Homogeneity of variance was analyzed using the Levene's test. Significant differences between treatments (*P*≤0.05) for the *in vitro* studies were determined by one-way ANOVA followed by Sidak's test. In case of unequal variances between treatments, the one-way ANOVA was followed by a Games–Howell test.

## References

[1] Janeway CA, Medzhitov R (2002). Innate immune recognition.. Annu Rev Immunol.

[2] Cannon JP, Dishaw LJ, Haire RN, Litman RT, Ostrov DA (2010). Recognition of additional roles for immunoglobulin domains in immune function.. Semin Immunol.

[3] Bork P, Holm L, Sander C (1994). The immunoglobulin fold. Structural classification, sequence patterns and common core.. J Mol Biol.

[4] Harpaz Y, Chothia C (1994). Many of the immunoglobulin superfamily domains in cell adhesion molecules and surface receptors belong to a new structural set which is close to that containing variable domains.. J Mol Biol.

[5] Barclay AN (1999). Ig-like domains: evolution from simple interaction molecules to sophisticated antigen recognition.. Proc Natl Acad Sci U S A.

[6] Du Pasquier L (2004). Speculations on the origin of the vertebrate immune system.. Immunol Lett.

[7] Martin AM, Kulski JK, Witt C, Pontarotti P, Christiansen FT (2002). Leukocyte Ig-like receptor complex (LRC) in mice and men.. Trends Immunol.

[8] Volz A, Wende H, Laun K, Ziegler A (2001). Genesis of the ILT/LIR/MIR clusters within the human leukocyte receptor complex.. Immunol Rev.

[9] Boyington JC, Brooks AG, Sun PD (2001). Structure of killer cell immunoglobulin-like receptors and their recognition of the class I MHC molecules.. Immunol Rev.

[10] Clemetson JM, Polgar J, Magnenat E, Wells TN, Clemetson KJ (1999). The platelet collagen receptor glycoprotein VI is a member of the immunoglobulin superfamily closely related to FcalphaR and the natural killer receptors.. J Biol Chem.

[11] Wines BD, Hulett MD, Jamieson GP, Trist HM, Spratt JM (1999). Identification of residues in the first domain of human Fc alpha receptor essential for interaction with IgA.. J Immunol.

[12] Pessino A, Sivori S, Bottino C, Malaspina A, Morelli L (1998). Molecular cloning of NKp46: a novel member of the immunoglobulin superfamily involved in triggering of natural cytotoxicity.. J Exp Med.

[13] Lebbink RJ, de Ruiter T, Verbrugge A, Bril WS, Meyaard L (2004). The mouse homologue of the leukocyte-associated Ig-like receptor-1 is an inhibitory receptor that recruits Src homology region 2-containing protein tyrosine phosphatase (SHP)-2, but not SHP-1.. J Immunol.

[14] Allcock RJ, Barrow AD, Forbes S, Beck S, Trowsdale J (2003). The human TREM gene cluster at 6p21.1 encodes both activating and inhibitory single IgV domain receptors and includes NKp44.. Eur J Immunol.

[15] Ford JW, McVicar DW (2009). TREM and TREM-like receptors in inflammation and disease.. Curr Opin Immunol.

[16] Clark GJ, Green BJ, Hart DN (2000). The CMRF-35H gene structure predicts for an independently expressed member of an ITIM/ITAM pair of molecules localized to human chromosome 17.. Tissue Antigens.

[17] Clark GJ, Cooper B, Fitzpatrick S, Green BJ, Hart DN (2001). The gene encoding the immunoregulatory signaling molecule CMRF-35A localized to human chromosome 17 in close proximity to other members of the CMRF-35 family.. Tissue Antigens.

[18] Clark GJ, Ju X, Tate C, Hart DN (2009). The CD300 family of molecules are evolutionarily significant regulators of leukocyte functions.. Trends Immunol.

[19] Diefenbach A, Raulet DH (2003). Innate immune recognition by stimulatory immunoreceptors.. Curr Opin Immunol.

[20] Moretta A, Bottino C, Vitale M, Pende D, Cantoni C (2001). Activating receptors and coreceptors involved in human natural killer cell-mediated cytolysis.. Annu Rev Immunol.

[21] Ravetch JV, Lanier LL (2000). Immune inhibitory receptors.. Science.

[22] Veillette A, Latour S, Davidson D (2002). Negative regulation of immunoreceptor signaling.. Annu Rev Immunol.

[23] Levine SJ (2004). Mechanisms of soluble cytokine receptor generation.. J Immunol.

[24] Torkar M, Haude A, Milne S, Beck S, Trowsdale J (2000). Arrangement of the ILT gene cluster: a common null allele of the ILT6 gene results from a 6.7-kbp deletion.. Eur J Immunol.

[25] Lebbink RJ, van den Berg MC, de Ruiter T, Raynal N, van Roon JA (2008). The soluble leukocyte-associated Ig-like receptor (LAIR)-2 antagonizes the collagen/LAIR-1 inhibitory immune interaction.. J Immunol.

[26] Gibot S, Kolopp-Sarda MN, Bene MC, Bollaert PE, Lozniewski A (2004). A soluble form of the triggering receptor expressed on myeloid cells-1 modulates the inflammatory response in murine sepsis.. J Exp Med.

[27] Schmid CD, Sautkulis LN, Danielson PE, Cooper J, Hasel KW (2002). Heterogeneous expression of the triggering receptor expressed on myeloid cells-2 on adult murine microglia.. J Neurochem.

[28] Gattis JL, Washington AV, Chisholm MM, Quigley L, Szyk A (2006). The structure of the extracellular domain of triggering receptor expressed on myeloid cells like transcript-1 and evidence for a naturally occurring soluble fragment.. J Biol Chem.

[29] Klesney-Tait J, Turnbull IR, Colonna M (2006). The TREM receptor family and signal integration.. Nat Immunol.

[30] Nikolaidis N, Klein J, Nei M (2005). Origin and evolution of the Ig-like domains present in mammalian leukocyte receptors: insights from chicken, frog, and fish homologues.. Immunogenetics.

[31] Yoder JA, Litman RT, Mueller MG, Desai S, Dobrinski KP (2004). Resolution of the novel immune-type receptor gene cluster in zebrafish.. Proc Natl Acad Sci U S A.

[32] Yoder JA (2009). Form, function and phylogenetics of NITRs in bony fish.. Dev Comp Immunol.

[33] Stet RJ, Hermsen T, Westphal AH, Jukes J, Engelsma M (2005). Novel immunoglobulin-like transcripts in teleost fish encode polymorphic receptors with cytoplasmic ITAM or ITIM and a new structural Ig domain similar to the natural cytotoxicity receptor NKp44.. Immunogenetics.

[34] Ostergaard AE, Martin SA, Wang T, Stet RJ, Secombes CJ (2009). Rainbow trout (Oncorhynchus mykiss) possess multiple novel immunoglobulin-like transcripts containing either an ITAM or ITIMs.. Dev Comp Immunol.

[35] Cannon JP, Haire RN, Mueller MG, Litman RT, Eason DD (2006). Ancient divergence of a complex family of immune-type receptor genes.. Immunogenetics.

[36] Dahm R, Geisler R (2006). Learning from small fry: the zebrafish as a genetic model organism for aquaculture fish species.. Mar Biotechnol (NY).

[37] Saeij JP, Van Muiswinkel WB, Groeneveld A, Wiegertjes GF (2002). Immune modulation by fish kinetoplastid parasites: a role for nitric oxide.. Parasitology.

[38] Saeij JP, de Vries BJ, Wiegertjes GF (2003). The immune response of carp to Trypanoplasma borreli: kinetics of immune gene expression and polyclonal lymphocyte activation.. Dev Comp Immunol.

[39] Buljan M, Bateman A (2009). The evolution of protein domain families.. Biochem Soc Trans.

[40] Litman GW, Hawke NA, Yoder JA (2001). Novel immune-type receptor genes.. Immunol Rev.

[41] Patthy L (1999). Genome evolution and the evolution of exon-shuffling–a review.. Gene.

[42] Long M (2001). Evolution of novel genes.. Curr Opin Genet Dev.

[43] Keren H, Lev-Maor G, Ast G (2010). Alternative splicing and evolution: diversification, exon definition and function.. Nat Rev Genet.

[44] Aguilar H, Alvarez-Errico D, Garcia-Montero AC, Orfao A, Sayos J (2004). Molecular characterization of a novel immune receptor restricted to the monocytic lineage.. J Immunol.

[45] Martinez-Barriocanal A, Sayos J (2006). Molecular and functional characterization of CD300b, a new activating immunoglobulin receptor able to transduce signals through two different pathways.. J Immunol.

[46] Ju X, Zenke M, Hart DN, Clark GJ (2008). CD300a/c regulate type I interferon and TNF-alpha secretion by human plasmacytoid dendritic cells stimulated with TLR7 and TLR9 ligands.. Blood.

[47] Clark GJ, Ju X, Azlan M, Tate C, Ding Y (2009). The CD300 molecules regulate monocyte and dendritic cell functions.. Immunobiology.

[48] Gamulin V, Rinkevich B, Schacke H, Kruse M, Muller IM (1994). Cell adhesion receptors and nuclear receptors are highly conserved from the lowest metazoa (marine sponges) to vertebrates.. Biol Chem Hoppe Seyler.

[49] Forlenza M, Scharsack JP, Kachamakova NM, Taverne-Thiele AJ, Rombout JH (2008). Differential contribution of neutrophilic granulocytes and macrophages to nitrosative stress in a host-parasite animal model.. Mol Immunol.

[50] Joerink M, Forlenza M, Ribeiro CM, de Vries BJ, Savelkoul HF (2006). Differential macrophage polarisation during parasitic infections in common carp (Cyprinus carpio L.).. Fish Shellfish Immunol.

[51] Heaney ML, Golde DW (1996). Soluble cytokine receptors.. Blood.

[52] Irnazarow I (1995). Genetic variability of Polish and Hungarian carp lines.. Aquaculture Research.

[53] Steinhagen D, Kruse P, Korting W (1989). The parasitemia of cloned Trypanoplasma borreli Laveran and Mesnil, 1901, in laboratory-infected common carp (Cyprinus carpio L.).. J Parasitol.

[54] Overath P, Ruoff J, Stierhof YD, Haag J, Tichy H (1998). Cultivation of bloodstream forms of Trypanosoma carassii, a common parasite of freshwater fish.. Parasitol Res.

[55] Steinhagen D, Hedderich W, Skouras A, Scharsack JP, Schuberth J (2000). *In vitro* cultivation of *Trypanoplasma borreli* (protozoa:kinetoplastida), a parasite from the blood of common carp *Cyprinus carpio*.. Dis Aquat Organ.

[56] Altschul SF, Gish W, Miller W, Myers EW, Lipman DJ (1990). Basic local alignment search tool.. J Mol Biol.

[57] Burge CB, Karlin S (1998). Finding the genes in genomic DNA.. Curr Opin Struct Biol.

[58] Pearson WR, Lipman DJ (1988). Improved tools for biological sequence comparison.. Proc Natl Acad Sci U S A.

[59] Thompson JD, Gibson TJ, Plewniak F, Jeanmougin F, Higgins DG (1997). The CLUSTAL_X windows interface: flexible strategies for multiple sequence alignment aided by quality analysis tools.. Nucleic Acids Res.

[60] Saitou N, Nei M (1987). The neighbor-joining method: a new method for reconstructing phylogenetic trees.. Mol Biol Evol.

[61] Page RD (1996). TreeView: an application to display phylogenetic trees on personal computers.. Comput Appl Biosci.

[62] Felsenstein J (1985). Confidence limits on phylogenies:An approach using the bootstrap.. Evolution.

[63] Joerink M, Ribeiro CM, Stet RJ, Hermsen T, Savelkoul HF (2006). Head kidney-derived macrophages of common carp (Cyprinus carpio L.) show plasticity and functional polarization upon differential stimulation.. J Immunol.

[64] Ribeiro CM, Hermsen T, Taverne-Thiele AJ, Savelkoul HF, Wiegertjes GF (2010). Evolution of recognition of ligands from Gram-positive bacteria: similarities and differences in the TLR2-mediated response between mammalian vertebrates and teleost fish.. J Immunol.

[65] Pfaffl MW (2001). A new mathematical model for relative quantification in real-time RT-PCR.. Nucleic Acids Res.

[66] Tichopad A, Dilger M, Schwarz G, Pfaffl MW (2003). Standardized determination of real-time PCR efficiency from a single reaction set-up.. Nucleic Acids Res.

[67] Weyts FAA, Rombout JHWM, Flik G, verburg-van-Kemenade LBM (1997). A common carp (Cyprinus carpio L.) leukocyte cell line shares morphological and functional characteristics with macrophages.. Fish Shellfish Immunol.

[68] Nakayasu C, Omori M, Hasegawa S, Kurata O, Okamoto N (1998). Production of a monoclonal antibody for carp (*Cyprinus carpio* L.) phagocytic cells and separation of the cells.. Fish Shellfish Immunol.

[69] Secombes CJ, van Groningen JJ, Egberts E (1983). Separation of lymphocyte subpopulations in carp Cyprinus carpio L. by monoclonal antibodies: immunohistochemical studies.. Immunology.

[70] Koumans-van Diepen JC, Egberts E, Peixoto BR, Taverne N, Rombout JH (1995). B cell and immunoglobulin heterogeneity in carp (Cyprinus carpio L.); an immuno(cyto)chemical study.. Dev Comp Immunol.

[71] Rombout JHWM, Koumans-van Diepen JCE, Emmer PM, Taverne-Thiele JJ, Taverne N (1996). Characterization of carp thrombocytes with specific monoclonal antibodies.. J Fish biology.

[72] Ribeiro CM, Hermsen T, Taverne-Thiele AJ, Savelkoul HF, Wiegertjes GF Evolution of recognition of ligands from Gram-positive bacteria: similarities and differences in the TLR2-mediated response between mammalian vertebrates and teleost fish.. J Immunol.

[73] Green LC, Wagner DA, Glogowski J, Skipper PL, Wishnok JS (1982). Analysis of nitrate, nitrite, and [15N]nitrate in biological fluids.. Anal Biochem.

